# Beware the Artifact: Generation of Ellagic Acid and Related Species During Atmospheric Solid Analysis Probe (ASAP) Measurements of Iron Gall Inks and Tannins in the Negative Ionization Mode

**DOI:** 10.1002/jms.70045

**Published:** 2026-03-25

**Authors:** Thomas Willms

**Affiliations:** ^1^ Bundesanstalt für Materialforschung und ‐prüfung (BAM) Berlin Germany

**Keywords:** ellagic acid, gallic acid, ink, metacyclophane, tannic acid

## Abstract

A detailed comparative analysis of the results of atmospheric solid analysis probe (ASAP) mass spectrometry of inks, tannins, and gallic acid (GA) in the negative ionization mode was performed. Results obtained for tannin by ASAP have been compared with those obtained with electrospray ionization (ESI). Apart from the GA ion, measurements frequently also yielded anions corresponding to association products and condensation products. At zero collision energy, pure GA gave a dimeric adduct (m/z 339); the formation of ions at m/z 303 and 304 was favored by increased temperatures and collision energies. In the case of inks, the subsequent dehydrogenation of such species to ellagic acid (EA) (m/z 301) was attributed to the oxidative effect of the iron III ion. On the basis of the results, a modification of the “standard ink protocol” has been suggested. Furthermore, several yet non‐described breakdown products and radical ion species of GA and EA ions were identified.

## 1 Introduction

Among the three main types of historical black writing inks, which are carbon ink, plant ink, and iron‐gall ink (or their mixtures), the use of the latter has emerged with the utilization of parchment as writing support in the early Middle Ages [1]. Iron gall ink was made from a source of gallic acid (GA), such as tannins (gall nuts, oak bark), soluble iron II ions (stemming from green vitriol (iron II sulphate) or other iron sources), and a water‐soluble binder (Arabic gum or gelatine) by subsequent oxidation to iron III. Historic manuscripts with black inks as writing materials have been mostly studied in the past by spectroscopic methods (RAMAN, IR, X‐ray fluorescence spectroscopy) [2, 3, 4, 5, 6, 7, 8], because such methods are noninvasive. In contrast, Hidalgo et al. [2] analyzed iron gall ink, as used in manuscripts, by HPLC with a diode array detector, RAMAN, and IR. Other authors combined chromatographic methods with mass spectrometry methods to study oak tannins [9, 10], ink hydrolysates [11], and the discoloration of organic red pigments [12]. Especially, the ions at m/z 169 and m/z 125 were utilized [3] to identify iron gall inks in manuscripts due to their content of gallic acid (GA). The possibility to identify tannins (tannic acid, TA) and their components, such as GA, in inks directly by ambient mass spectrometry (AMS) methods was studied only recently [2, 3]. Such methods originate from atmospheric pressure ionization (API) techniques [13, 14, 15, 16]. While they are not noninvasive, they are highly sensitive and require only small quantities for analysis. Therefore, they can be considered minimally invasive. AMS techniques, such as the atmospheric solids analysis probe (ASAP) and electrospray ionization (ESI), have revolutionized the field of mass spectrometry by allowing the direct ionization of analytes in complex samples under atmospheric pressure conditions [13, 17]. The ASAP method is particularly advantageous for the rapid and versatile ionization and direct analysis of volatile and semi‐volatile liquid and solid samples (such as polymers, tissues, and powders), without the need for extensive sample preparation [18, 19] or chromatography. ASAP operates by applying a high voltage to a sample brought into contact with a heated glass probe, leading to rapid desorption and vaporization of analytes from the probe. This generates an ion stream that is introduced into the mass spectrometer. ASAP is most appropriate for molecule masses below 1000 Da and medium‐polar compounds [13], because samples need to be volatile enough. Using the negative ionization mode, ASAP has been applied to identify the organic compounds of inks and distinguish different ink types [3].

**FIGURE 1:**
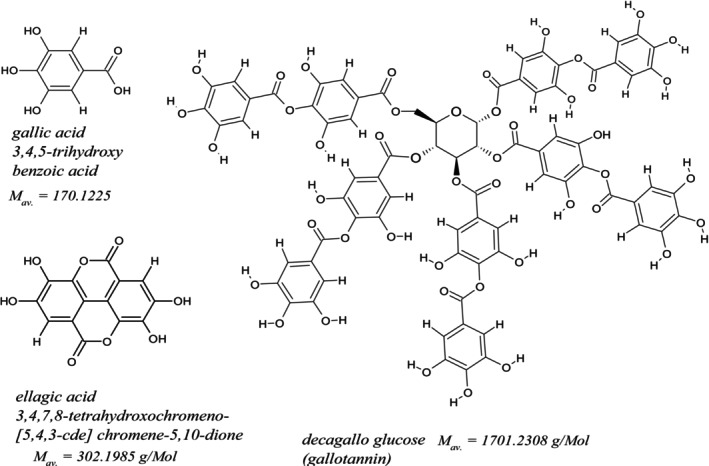
Structures of GA, EA, and decagallo glucose (TA) including average mol masses.

“Tannin”, the main component of iron‐gall inks, is not a single well‐defined substance but comprises many different compounds. As an example for gallotannins, used in this work, a decagallo tannin is shown in Figure 1. However, commercial tannin products (TA) can contain different compounds of this type and, in part, also impurities such as GA and EA. Therefore, the presence of ellagic acid (EA; Figure 1) in TA could not be ruled out, and commercially available TA was analyzed by ESI [20, 21, 22, 23] to assure the absence of EA in TA. ASAP measurements of mock ink samples, produced from TA, in negative ionization mode frequently revealed signals for EA [3]. For gall inks produced from GA, this was not observed. However, the formation of EA from similar gall ink by autoxidation after 6 months of ageing [24] was reported in 2022, whereas freshly prepared inks did not yield EA. Furthermore, the formation of other so‐called aging markers, such as digallic acids, was observed at low air humidity. Notably, Melo et al. [25] did not mention the formation of EA or other ageing markers in their review on ink degradation in 2022.

In this work – in view of the aging effect – freshly prepared mock samples of iron‐gall inks, TA, and GA have been analyzed by ASAP, varying the experimental conditions of the ASAP measurement, to study the formed species. For a comparison of the spectra and identification, also, EA itself was measured. For ESI, it is known that because of electrochemical reactions [23], oxidation of the analytes can occur. Because the fragmentation of analytes into ions with an odd electron number, such as observed with ASAP and ESI measurements, has been little investigated [21] and remains poorly understood, their formation was also studied. The results of ASAP measurements of inks, TA, and GA under various conditions were compared to determine whether the detection of EA under such conditions can be clearly associated with the presence of EA in the writing material.

## 2 Experimental Section

In this work, a time‐of‐flight (TOF) mass spectrometer Xevo QToF G2 SX (Waters, Manchester, United Kingdom) was used, equipped either with an ASAP or alternatively with an ESI probe (Waters, Manchester, United Kingdom). All measurements and calibrations were performed in the negative ionization mode using the sensitivity mode with a resolution of ~22 000. Only peaks within the linear dynamic range of the MS detector (~10^3^–10^8^) were considered for evaluations of the isotopic peak intensities. For the ASAP measurements, glass capillaries (X100 capillary tubes sealed O.D. 1.8/2 mm, Fisher Scientific) were utilized. The ESI method was utilized for sample analysis and also to calibrate the mass spectrometer. All ASAP and ESI measurements (Table A1) and calibrations were performed using the software MassLynx (Waters) (see Section 2.3).

Calibration of the mass spectrometer was performed regularly with a 0.1% sodium formate solution. The sodium formate solution was prepared from sodium hydroxide (99.99%, VWR International), formic acid (100%, Optima, Fisher Scientific), acetonitrile (99%, Carl Roth GmbH + Co. KG), and ultrapure water (Table 1). All chemicals were of MS grade. Preparation followed the protocol provided by Waters. The solution is typically applied for ions with m/z values ranging from 100 to 1200. In this work, however, it was employed for calibration over an extended range from m/z 50 up to 2000, applying a higher sample cone voltage of ≥ 100 V instead of 30 V.

**TABLE 1:** Substances used for the analysis and the production of inks.

Substance	Amount used for ink, g	Purity, %	Average mol mass, g/mol	Supplier
Arabic gum	0.785	Purified	~2.5E4	Kremer Pigmente^a^
ES	—	> 90	302.197	Carl Roth, GmbH + Co. KG
GA	0.17	> 99	170.022	
Ultrapure water	(see text)	99.9	18.0153	
Iron (II) sulfate heptahydrate	1.05	99	278.01	Sigma Aldrich AG
TA^c^	1.17^b^	> 99	1701.23	

^a^ 63 300.

^b^ Instead of 1.23 g TA (95%) [26] due to higher purity.

^c^ Article 403040 (PhE).

For continuous mass‐axis correction during data acquisition, regularly, a lock mass solution was prepared according to the recommendations of Waters using the pentapeptide leucine–enkephaline (Leu–Enk, Tyr–Gly–Gly–Phe–Leu, CHNO) as standard reference (Waters, #186006013). The solution that was introduced via the dedicated lockspray channel provided a stable reference ion at m/z 554.2615 with its satellite signals, which were monitored throughout each run, enabling real‐time correction of minor mass drift and ensuring consistent mass accuracy across all analyses.

### 2.1 Fabrication of Inks

Ferrous sulfate (iron II sulfate), EA, GA, and TA and demineralized water were used as received (see Table 2 for details). Mock ink samples were prepared from an aqueous solution of iron sulfate and Arabic gum (10 mL), and either TA or GA in 25 mL water (to obtain either TA ink or GA ink), according to Neevel [26], who provided a widely utilized “standard protocol” (based on many historical samples), respectively, using the substance amounts specified in Table 1. The solutions were then combined, and the resulting ink was applied to Whatman paper with a bamboo stick.

**TABLE 2:** Signals obtained with ASAP measurements of GA ink, TA ink, and TA samples in the negative ionization mode (for details of experiments, see the number of the experiment (Exp.) in Table A1), and their interpretation (RA: radical anion), in the order of the monoisotopic ions, with their minor isotope signals and theoretical relative intensities.

m/z		Δm/z		Interpretation [26, 27, 28]	
Obs.	Calc.^a^	mDa	Assignment	Elemental composition	Exp.
169.0142	169.0137	0.5	170 − 1p	C_7_H_5_O_5_: GA^−^ = GA–H^+^ (GA anion)	1A
170.0170	170.0176	−0.6	169 + 1n	C_7_H_5_O_5_: GA^−^: first isotopic peak (7.8%)	1A
171.0190	171.0210	−2.0	169 + 2n	C_7_H_5_O_5_: GA^−^: second isotopic peak (1.3%)	1A
172.0199	172.0243	−4.4	169 + 3n	C_7_H_5_O_5_: GA^−^: third isotopic peak (0.1%)	1A
299.9906	299.9906	0.0	300e = 302 − H_2_ + e	C_14_H_4_O_8_: RA‐300: EA–H_2_ + e^−^	1D
301.0010	300.9945	6.5	300e + 1n	C_14_H_4_O_8_: RA‐300: first isotopic peak (15.5%)	1D
302.0078	301.9979	9.9	300e + 2n	C_14_H_4_O_8_: RA‐300: second isotopic peak (2.8%)	1D
301.0010	300.9984	2.6	2 · 170–2 · 18–2–1p	C_14_H_5_O_8_: EA^−^ = 2 GA–2 H_2_O–H_2_–H^+^	1C, 1D
302.0078	302.0023	5.5	301 + 1n	C_14_H_5_O_8_: EA^−^: first isotopic peak (15.5%)	1C, 1D
303.0049	303.0057	−0.8	301 + 2n	C_14_H_5_O_8_: EA^−^: second isotopic peak (2.8%)	1C, 1D
304.0238	304.0091	14.7	301 + 3n	C_14_H_5_O_8_: EA^−^: third isotopic peak (0.3%)	1C, 1D
302.0078	302.0063	1.5	302e = 302 + 1e	C_14_H_6_O_8_: RA‐EA: 2 GA–2 H_2_O–H_2_	1D
303.0049	303.0102	−5.3	302e + 1n	C_14_H_6_O_8_: RA‐EA: first isotopic peak (15.5%)	1D
304.0149	304.0135	1.4	302e + 2n	C_14_H_6_O_8_: RA‐EA: second isotopic peak (2.8%)	1D
303.0129	303.0141	−1.2	2·170–2 · 18–p	C_14_H_7_O_8_: MP^−^: 2 GA–2 H_2_O–H^+^	1D, 1E
304.0206	304.0175	3.1	303 + 1n	C_14_H_7_O_8_: MP^−^: first isotopic peak (15.6%)	1D, 1E
305.0238	305.0208	3.0	303 + 2n	C_14_H_7_O_8_: MP^−^: second isotopic peak (2.8%)	1D, 1E
304.0218	304.0219	−0.1	2 · 170 − 2·18 + e	C_14_H_8_O_8_: RA‐MP^−^: second GA − 2 H_2_O	1E, 1F
305.0238	305.0258	−2.0	304 + 1n	C_14_H_8_O_8_: RA‐MP^−^: first isotopic peak (15.6%)	1E, 1F
306.0234	306.0292	−5.8	304 + 2n	C_14_H_8_O_8_: RA‐MP^−^: second isotopic peak (2.8%)	1E,1F
307.0302	307.0325	−2.3	304 + 3n	C_14_H_8_O_8_: RA‐MP: third isotopic peak (0.3%)	1E,1F

^a^ The given values are provided by MassLynx. They do not include the electron mass *m*_*e*_.

### 2.2 Reproducibility and Repeatability

Because a precise quantitative reproduction of the signal intensities of ASAP measurements was not possible (slightly better for liquid samples than for powder samples), due to the very small quantities (< 1 μg ink sample), different experiments cannot be quantitatively compared. However, the qualitative repeatability and reproducibility of the intensities were acceptable. The reproducibility depended mainly on the quality of the calibration and the internal correction by the lock mass standard. The m/z value of GA, determined by different operators, calibrations, and methods, was measured at m/z 169.0120 ± 0.0050, the correct value being m/z 169.0142. For EA, the measured m/z values were m/z 302.0030 ± 0.0050, the correct value being 302.0063. Repeatabilities for the same method and calibration were better and gave confidence intervals of ± 2 mDa. The selected results have been observed in several similar experiments by different operators and also under slightly varying conditions.

It must be indicated, however, that the qualitative reproducibility in case of ink measurements was very limited, which is in part subject of this article.

### 2.3 Evaluation of Experiments

Experiments were evaluated using the MassLynx 4.2 (Waters) tool “Elemental composition.” It permitted the identification of the elemental composition of the species, using the difference to the calculated m/z value, the plausibility (meaningful formula, possible product) of the chemical formula, and the fit confidence (%) being a measure of how well the isotopic pattern matches the theoretical model. Depending on the signal intensity under consideration, the fit confidence varied significantly, and the identification of some compounds was frequently hampered by the presence of additional peaks. Where relevant, these issues are discussed (see also Supporting Information: Sections 1.2 (calculations) and 1.4 (identification)).

## 3 Results

Because iron gall inks do not dissolve in water, the ESI method cannot be used for their analysis. Therefore, ASAP measurements were used to determine the type of ink, used for manuscripts [1, 3]. For each result, represented in a figure or in a table, the experimental conditions of the method have been summarized in Table A1 (Chapter 7, Appendix) with the abbreviation as given in the text and in the last column of the tables (e.g., 1A, 2).

### 3.1 ASAP Measurements

Despite multiple experiments, performed under various conditions, such as lower temperatures, corona currents, and sample cone voltages, it was not possible to detect ions corresponding to the intact TA molecule (monoisotopic mass of 1700.1730 g/mol) or larger fragments thereof by ASAP experiments. Only ion masses below m/z 310 were observed, predominantly at m/z 169, which corresponds to the anion of GA (monoisotopic mol mass of 170.0215 g/mol).

In negative ionization mode, generally, a lower fragmentation degree of the analytes was observed than in the positive mode [27]. Therefore, in mass spectra of GA, measured in the negative mode, the signal belonging to the anion of GA at m/z 169 was frequently prominent, although to a minor extent also the ion at m/z 125, the decarboxylation product pyrogallol [3] was observed depending on the reaction conditions. Those ions, as well as a signal at m/z 301, due to the content of EA, are typical for iron gall inks and TA and were utilized to detect the latter on manuscripts or mock samples. Because the original composition of historic inks is generally unknown, freshly prepared homemade inks were used (see Section 2) to study the influence of the ASAP measurement conditions on the resulting mass spectra of inks. Historic inks are generally more similar to TA inks, but an ink with GA is better defined than TA inks because the exact composition of commercial “tannin” (without further specification) is complex. On the packaging of tannin, generally, only a theoretical formula from literature, decagallo glucose (Figure 1), was provided; the actual composition remained unknown (see Supporting Information: Section 2). In the following, ASAP measurements with GA ink, TA ink, TA, and GA are analyzed and compared.

### 3.2 Measurement of GA Inks in the Negative Ionization Mode

**FIGURE 2:**
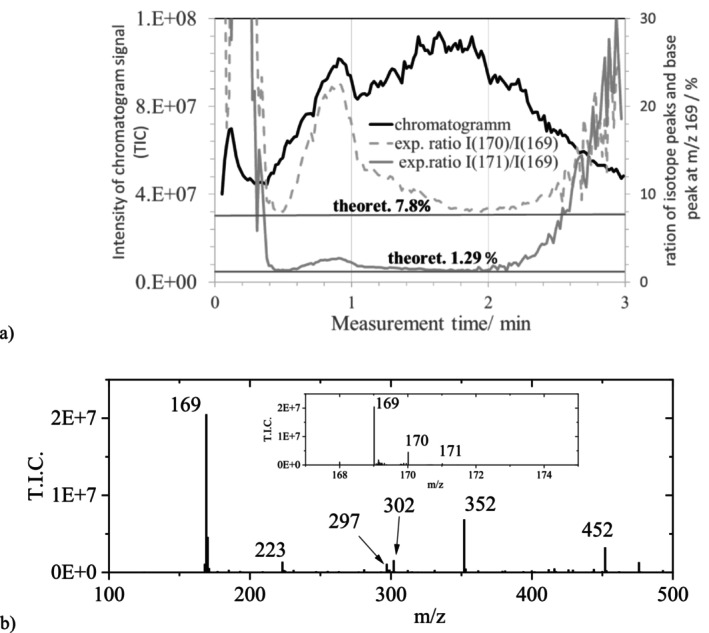
ASAP measurement of GA ink 1 measured in the negative mode (Table A1, Exp. 1A). (a) Chromatogram (left *Y*‐axis) and traces of the ratios of the relative intensities I of minor isotopic peaks (m/z 170 and 171) and m/z 169 (right *Y*‐axis), and their theoretical values (lines). (b) Mass spectrum acquired at the maximum of the GA peak (0.8 min) from the chromatogram with the gallate ion at m/z 169, background signals, and a magnification of the minor isotopic peaks of the gallate ion.

The ASAP experiment (Exp.) with a sample of GA ink 1 (GA ink, see Section 2), measured at a constant probe temperature of 650°C and lower sample cone voltage (30 V) (Table A1, Exp. 1A), yielded a chromatogram (Figure 2a) with a broad peak, ranging from 0.5 to 3 min, with maxima at 0.9 and 1.6 min. The maximum of the signal at m/z 169.0113 in the corresponding mass spectra (Figure 2b) was obtained at 0.9 min measurement time with an intensity of I(169) = ~8.5E7. Due to the high resolution of the MS (~22 000), the signal at m/z 169, assigned to GA, could be clearly separated from a smaller neighboring background signal, present at m/z 168.9796, with an intensity I < 5E5 (I(168.9796) < 2%).

Using the tool “Elemental composition” from MassLynx (Section 2.3), which is based on the precise ion mass and the isotopic pattern (i.e., the ratios of the first and second isotopic peaks), the ion at m/z 169.0090 in the mass spectrum Figure 2b, at a retention time of 1.8 min in the ASAP measurement, was attributed to the composition C_7_H_5_O_5_ with a fit confidence of 100%, corresponding to the composition of the GA anion (m/z 169.0137; see Supporting Information: Section 1.3). Figure 2 shows that, at the corresponding higher measurement times, the relative intensity ratios of the minor isotopic peaks of 8.2% and 1.3% match quite well the corresponding theoretical values of 7.8% and 1.3% [28, 29, 30], respectively (assuming a 1.07% ^13^C content, for details see Supporting Information: Section 1.1).

However, in the same measurement at a measurement time of 0.87 min, MassLynx yielded only a fit confidence of 71.5% for the composition corresponding to GA. An analysis of the minor isotopic peaks of the GA signal revealed that this was caused by a significant deviation of the relative intensities at m/z 170 (containing one ^13^C) and at 171 (containing two ^13^C) from the theoretically calculated values (Figure 2). The deviation is unlikely to be due to isotope discrimination effects [31], because the presence of the latter, for example, during a vaporization, should lead to a higher abundance of lighter isotopes at lower and of heavier isotopes at higher measurement times (or at higher temperatures if ramp) in the gas phase. Furthermore, such isotope discrimination effects or transmission field effects in the MS may lead to intensity ratio error of about 5% (as observed in Figure 2) up to 10% but not to errors of 100%. Because the intensities of both isotopologues changed in parallel, the observed deviations (at m/z 170 and m/z 171) were tentatively attributed to another species, formed during the measurement. This is further analyzed and discussed in Section 4. Thus, the ASAP measurement of GA ink 1 (Figure 2), using the standard method 1 (Table A1), only indicated the presence of GA and another species at m/z 170, whereas no signals of reaction products of GA at higher m/z values were observed, for example, the signals at m/z 301.9806 and 351.9782 belonging to the background. Similar mass spectra as for GA ink 1 were obtained for other GA inks, not containing iron (Table A1, Exp. 1B). The identification of GA via MassLynx with a fit confidence of 100% was only possible at the beginning and at the end of the GA peak; at the maximum only, a fit confidence of 20% was obtained for GA. In the range m/z 300 to 304, no product peaks were observed, in contrast to tannin inks that are considered in the following section.

### 3.3 Measurement of Tannin Inks

#### 3.3.1 Measurement of TA Ink 1

**FIGURE 3:**
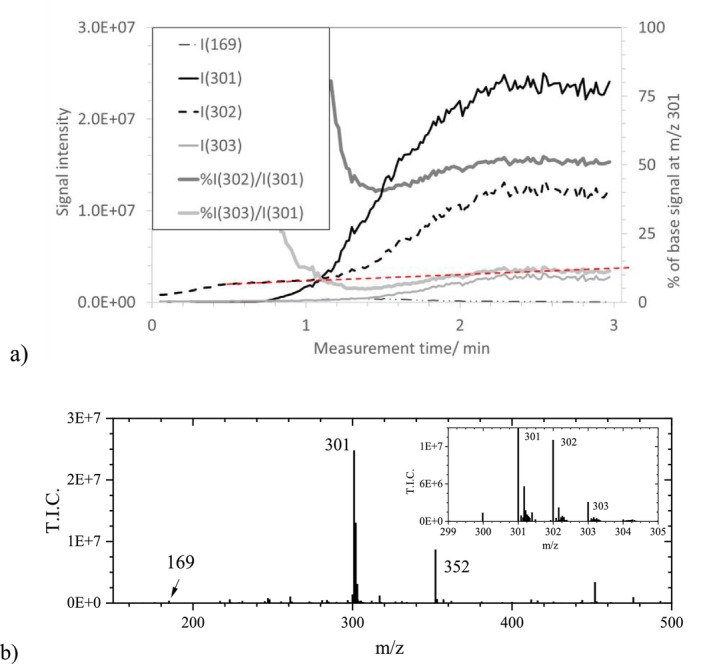
(a) Signal intensity courses of GA and EA anions in the chromatogram of an ASAP measurement of a TA ink 1 (Table A1, Exp. 1C) obtained with the standard method (Table A1, Exp. 1C). The red line indicates the estimated course of the intensity of the background signal at m/z 301.9806, which merges with the first minor isotope signal of EA at m/z 301.9990. (b) Mass spectrum (and magnified isotopic peaks) at 2.8 min of this ASAP measurement.

An ASAP measurement of a sample of the homemade TA ink (“TA ink 1”) (Section 2), using the same method (Table A1, Exp. 1C) as for GA ink 1, yielded a chromatogram with an increasing baseline and a hardly recognizable maximum at the end of the measurement. One base signal at m/z 301.0003 with minor isotopic peaks at m/z 302 and 303 (Figure 3b) dominated the spectra; the peak at m/z 169.0078 at ~1.2 min was 500 times smaller than the base signal. A certain difference in composition between the GA ink and the TA ink is the higher GA content of the latter, since, assuming the structure of a decagallo tannin (see formula in Figure 1), 1 mol TA contained 10 mol GA units (which might form GA by decomposition). Therefore, using 1.17 g TA per 35 mL water (Table 1), the corresponding amount of 6.88E−4 mol TA could result in approximately 6.88E−3 mol GA by decomposition, which is more than that is present in the solution for the GA ink (0.17 g = 1E‐3 mol GA per 35 mL water). Using about 2 μL solution for both ASAP measurements, this might contribute to the formation of additional products at higher m/z values than 301, but this difference is likely not decisive, since in both cases, the GA quantities were of the same order of magnitude.

Although the signal at m/z 301 was intensive and the m/z value corresponded well to that calculated for EA (m/z 300.9989) by MassLynx, only a maximum fit confidence of 25% was obtained for the formula C_14_H_5_O_8_ EA^−^ (based on a natural ^13^C abundance of 1.07%; see Supporting Information: Section 1.1). Indeed, Figure 3a shows that the relative intensities of the signals at m/z 302 and at m/z 303 were about 50% and 15% of the monoisotopic peak, which is significantly higher than the calculated ion intensity ratios (15.48% and 2.76%). This explains the low fit confidence for EA (Supporting Information: Section 1.3) and indicates that the observed intensities are altered by the presence of another species, which will be discussed later (Section 4.2; Figure 12). In some spectra, only ~1% was attributed to EA and a fit confidence above 99% was assigned to the composition C_17_H_1_O_6_. Such a misassignment can occur when the isotopic pattern does not correspond to the calculated minor isotopic peak intensities of EA at m/z 302 (15.5%) and 303 (2.8%).

**FIGURE 4:**
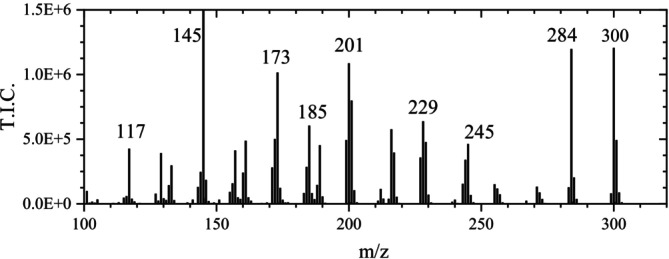
Mass spectrum obtained from an MSMS–ASAP measurement of an ink A 4 sample in the negative ionization mode (Table A1, Exp. 2).

To ensure that the observed ion at m/z 301 corresponds to the EA anion, as it was likely, additional investigations were carried out. To identify EA, an ink sample was studied by an MSMS experiment with a higher collision energy of 35 eV, focusing on m/z 301 (Figure 4). In this case, the peak at m/z 301 was reduced to what might be the minor isotopic peak of the species at m/z 300. The latter was among the highest peaks in the mass spectrum, but many additional signals below m/z 300 were also present.

A comparison of the mass spectrum of the ink sample (Figure 4) with that of EA (Figure 11) and literature data showed that signals at m/z 284, 245, 229, and 185 are typical fragment ions of EA (see section 3.6). Differing intensities should be due to the higher collision energy. Thus, the peak at m/z 301 must be due to EA, despite the small fit confidence. Moreover, the mass spectrum (Figure 11) shows that the signal at m/z 300 is a fragment ion of the monoisotopic signal of EA (m/z 301).

Furthermore, the traces of the peaks 301, 302, and 303 were compared with that of GA at m/z 169 (magnified; Figure 3). Under the given conditions, the peak at m/z ~ 301 approximately began to increase, starting at ~1 min, where the signal of GA at m/z 169 already reached its maximum. Accompanied by a decrease in the GA signal, the peak increased approximately exponentially over the measurement time. This observation would be consistent with a formation of EA from GA and a vaporization temperature above 300°C. However, precise vaporization data were not available [32] due to thermal decomposition. The higher vaporization temperature might have been the reason why Arpini et al. [11]—measuring in the positive mode up to 290°C—could not detect it.

#### 3.3.2 Measurement of TA Ink 2

**FIGURE 5:**
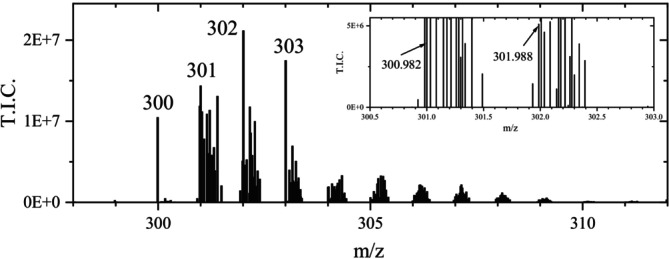
Mass spectrum obtained at a measurement time of 2.9 min from an ASAP measurement of the TA ink 2 sample in the negative ionization mode (Table A1, Exp. 1D) with magnified section, indicating background signals.

The ASAP measurement of another sample of the same TA ink (“TA ink 2”) also resulted only in a small signal for GA (m/z 169). Whereas GA was nearly completely consumed, additional signals appeared in the region between m/z 300 to 307 (Figure 5).

At the beginning of the measurement (below 1 min), there was a signal at m/z 300.9950, which was identified by “Elemental composition” with a fit confidence of 33% as EA. However, this was a background signal that could be distinguished from EA by the absence of an isotopic peak at m/z 302. The misassignment was due to two small background signals, which were present at m/z 301.9806 (see Figure 2, m/z 302) and at m/z 302.9966. In contrast to this, at measurement times above 1.6 min, the signal of EA at m/z 300.9985 with all isotopic peaks was present (Figure 5). However, there were other signal interferences. In the case of this ink sample, the peak at m/z 300 was found to be of comparable height with that at m/z 301, whereas in Exp. 1C, it was considerably smaller than that at m/z 301. According to calculations (Table 2), only minor isotope signals with relative intensities of 15.5% and 2.8% were expected at m/z 302 and 303, corresponding to the presence of one and two ^13^C atoms, respectively [28, 29, 30]. However, in this case, the signals at m/z 302 and 303 were significantly higher than that at m/z 301, although, given the small molecule of EA, the minor isotopic peaks cannot exceed the intensity of the monoisotopic peak, which is only observed for molecules larger than C_90_ [31]. Thus, the signals at m/z 302 and 303 cannot be due solely to the minor isotopic peaks of the signal at m/z 301. This also indicates that the increased relative intensities at m/z 302 and m/z 303, observed for the sample TA ink 1, should be due to other species, similar to EA. Also, in this case, the composition of the latter (C_14_H_5_O_6_) was attributed to the ion m/z 301 by MassLynx only with a fit confidence below 25%. Furthermore, the chemically most likely composition C_14_H_6_O_6_ for the signal at m/z 302 yielded only a very small fit confidence in this measurement (< 1%). The compositions C_17_H_1_O_6_ and C_10_H_7_O_11_, respectively, suggested by MassLynx for m/z 301 and 302, both with a fit confidence above 90%, were considered as chemically implausible and caused by a misassignment (see Supporting Information: Section 1.3). This has already been shown for GA in this work and is further demonstrated by ASAP measurements with EA (Section 3.6), which revealed the same issue.

For the given ASAP measurement, samples of the same ink batch were analyzed, using the same sampling and measurement method. Thus, identical signals at the same m/z values were expected, but a comparison of Figures 3b and 5 shows that this was not the case. The differences are explained in Section 4. The ion at m/z 302 was tentatively interpreted as the radical anion of EA. However, due to the suggestion of chemically meaningless compositions, as explained before for EA, this anion could not be unambiguously identified by MassLynx.

#### 3.3.3 EA Content of TA

Although it was not very likely that EA was already contained in the TA used for the preparation of the ink at a significant amount, this possibility had to be excluded. Considering the intensity of the EA peak, which was typically 500 times higher than that of GA in the same mass spectrum, the composition of the used TA was further investigated by ESI. Such a high signal intensity for EA would suggest that EA was present in much higher quantities than GA in the TA. However, although commercial TA is not a uniform substance, such a high content is not plausible because a gallotannin was used for the ink production.

**FIGURE 6:**
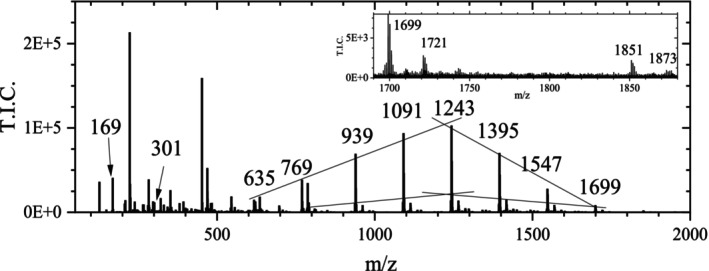
Mass spectrum obtained from an ESI measurement of TA in the negative ionization mode (Table A1, Exp. 9), with magnification of the highest ion masses, showing the typical isotopic pattern of the signals (with m/z values).

In the mass spectra from the ESI measurements of TA in this work (Figure 6, see also Supporting Information: Section 2), the EA signal at m/z 301 was nearly undetectable and reached only an intensity of *I*: ~1E4, whereas the sum of the intensities of all signals (at m/z 787 up to m/z 1699, with increments of 152 u), originating from gallotannin fragments, was at least *I*_total_ (tannin fragments) = 4.37E05.

The highest peaks, assigned to gallotannin, exhibited an intensity up to ten times higher, while the two smallest peaks had intensities equal to that of the peak of EA at m/z 301. Considering the higher signal of GA at m/z 169 (*I*: 4E4) and the intensity of the TA signals, such a small EA signal should correspond to a negligible quantity of EA.

An evaluation of the relative intensities of the signals at m/z 301 (0.16% of total intensity), corresponding to the content of EA, and those corresponding to TA (6.2% of total intensity) of the ESI measurement (Figure 6) showed that the EA was during the whole ASAP measurement too small to cause the important signals obtained with TA and TA inks in ASAP measurements.

In contrast to the mass spectra of the ASAP measurement of TA ink 2, the intensities of the peaks at m/z 302, 303, and 304 in the ESI mass spectra were much smaller than that at m/z 301, corresponding to the expected intensities of the minor isotopic peaks (theoretically ~15.48%, ~2.76%, and ~0.30%). The spectrum of Fu et al. [21], measured in the positive mode with addition of potassium ions, is not directly comparable with the measurements of this work, but it also did not indicate a significant content of EA in commercial gallotannin.

Thus, although EA should not have been present in the case of TA ink samples, the standard method gave in part only a signal of EA, in part signals of other species, whereas this did not happen with GA ink. Therefore, there might be an influence of other parts of the TA molecule. To eliminate first any influence of the iron cation, which leads to oxidation of the EA molecule, measurements have been performed with TA itself.

### 3.4 Tannin

**FIGURE 7:**
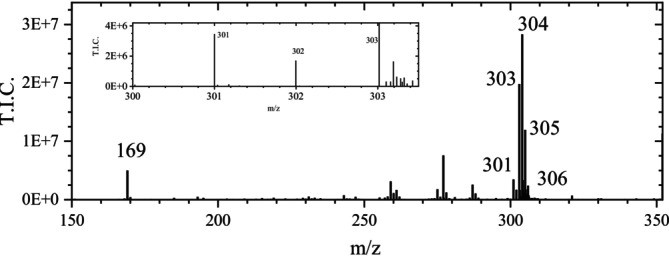
Mass spectrum obtained from the ASAP measurement of a TA solution ([TA]_0_: 4.6E−4 mM) (Table A1, Exp. 1E) at 2.868 min, measured in the negative ionization mode.

Using samples of pure TA, no iron ions and also no Arabic gum influenced the measured product signals, in contrast to inks. However, in the case of dry samples, the sample mass was difficult to control. Therefore, first, the influence of the concentration of aqueous TA solutions on the signal was studied, and the TA quantity was estimated. When 2 μL of the undiluted solution ([TA]_0_: 2.3 E−3 mM) was used for the ASAP measurement, a maximum amount of approximately 4.6 pmol of TA could be present on the glass rod. For simplicity, assuming a pure decagallotannin, this corresponds to a maximum of 46 pmol or 7.8 ng GA. Utilizing the same conditions as for TA ink 1 (Figure 3; Table A1, Exp. 1C), the undiluted aqueous TA solution ([TA]_0_) produced a large signal in the chromatogram at *t* = 1.2 min (Exp. 1D), with a prominent signal at m/z 169 in the mass spectra at low retention times. At higher retention times, signals in the range of m/z 301 to 307 got more significant, except for the most diluted TA solution (100×), corresponding to an amount of only 78 pg GA. A typical mass spectrum at a retention time of approximately 2.87 min is shown in Figure 7. The signal at m/z 304 was five times more intense than that at m/z 169 and, based on the m/z value 304 and the fit confidence of 99 to 100% in most spectra, could be unambiguously assigned with MassLynx to the composition C_14_H_8_O_8_.

The signal of GA measured at m/z 169.0140 was attributed to GA with a fit confidence of 100% and corresponds to the correct value of m/z 169.0142 although MassLynx gave an error of 0.3 mDa. In this case, this is due to a systematic error of MassLynx, which does not include the mass of the electron (see Supporting Information: Section 1.1) and calculated only a value of m/z 169.0137.

**FIGURE 8:**
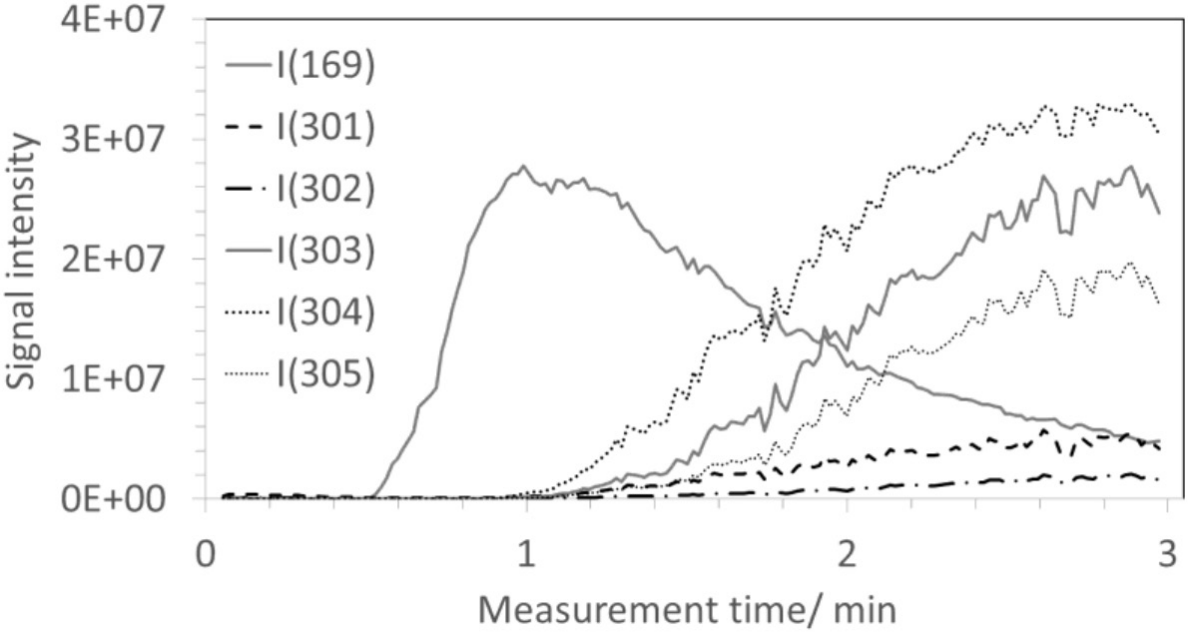
Chromatogram of the ASAP measurement of 2 μL of a 2.3E−4 mM TA solution measured in the negative ionization mode (Table A1, Exp. 1F) with typical courses of the signal intensities at m/z 169 (without magnification), 301, 302, 303, and 304.

Using the same method and varying the dilution of the TA solution (5, 6, 7, 8, 10, 100×) (Table A1, Exp. 1F), the signal traces of the ions at m/z 301 to 304 in the ASAP chromatograms remained mostly unchanged. As an example, the traces of the ions at m/z 301 and 169 for a 10 times diluted TA solution are shown in Figure 8. Once again, the expected minor isotopic peaks of EA at m/z 303 and 304, with calculated relative intensities of 15.48% and 2.76%, respectively (Table 2), should be much less intense than the monoisotopic peak in the case of such a small molecule. Because the signals at m/z 303 and 304 were much more intense than the expected minor isotopic peaks of the peak at m/z 301, they must correspond to ions of other—yet unknown—species. Based on this assumption and disregarding the peak at m/z 301 due to its low intensity, the signal heights observed at m/z 305, 306, and 307 should represent the sums of the minor isotopic peaks arising from both the species at m/z 303 and 304.

However, using relative intensity values of 15.48% for the first minor isotopic peak and 2.76% for the second minor isotopic peak for both signals at m/z 303 and 304 did not account for the intensities of the signals at m/z 305 and 306, as these were both considerably higher than the calculated sums of the theoretical minor isotopic peaks intensities, 2E07 instead of 5E06 at m/z 305 and 5.6E06 instead of 9.4E05 at m/z 306. The reason for this discrepancy is unknown.

The observed peaks at m/z 303 and 304 indicate that, in addition to the ionization of GA present in the TA sample and leading to a signal at m/z 169, further processes occurred. Thus, besides traces of EA, two additional negatively charged species with prominent signals at m/z 303 and 304 were detected (Figure 8). The origin of additional peaks, appearing at non‐integer m/z values, is unknown, but might be due to fragments related to glucose or impurities of the used tannin. The small content of EA in TA, detected by ESI measurements (Supporting Information: Section 2), appeared to be unaffected in the case of the TA measurement (Figure 8).

In a further ASAP experiment (Table A1, Exp. 1G), adding 1 mmol Arabic gum to the TA solution, the signal courses were only slightly different. In contrast to Figure 7, the signal at m/z 169 reached nearly half the intensity of the peak m/z 304 at the same retention time.

In the given mass spectrum, the anion m/z 303 was identified with MassLynx as an anion with an even electron number, the composition C_14_H_7_O_8_, and a fit confidence of 57%. The missing 40% of fit confidence was attributed to a compound with the composition C_7_H_11_O_13_ due to a misassignment (Supporting Information: Section 1.3) (as in the case of EA). Thus, the result is a significant indication for an anionic species at m/z 303 of which the neutral molecule (non‐observable) has the molecular weight of 304 g/mol. From the latter, the anion at m/z 304 could have been formed, which was identified by MassLynx as a species anion with an even number of hydrogen atoms and an odd number of electrons (composition: C_14_H_8_O_8_) in the ASAP measurements of this work. Applying the same ASAP method, a pronounced signal at m/z 304 was observed for TA samples, but not for the TA ink samples. An overview of the foregoing results is given in Table 2.

In the case of both TA ink measurements, the signal at m/z 304 was entirely or mostly absent in the mass spectra. The results indicate that other components of TA influenced the ASAP measurement, as has also been suspected for the TA ink samples. The formation of the corresponding intermediate product (MP) will be rationalized further in Section 4.2. Furthermore, in the case of the TA ink, the observed EA signal could not be due to a content of EA of the TA utilized for its production. Because EA and similar products observed with TA inks and TA are due to a reaction of GA, contained in TA, the reaction will be studied using a GA sample (Section 3.5). Therefore, ASAP measurements with GA have been performed.

### 3.5 GA

Utilizing other conditions than the standard method, apart from m/z 169 and m/z 125 [3], further species were observed. Under some reaction conditions, the signal at m/z 169 was also absent in the mass spectrum of the ASAP measurement.

#### 3.5.1 Measurements of GA Solutions

Because 2 μL of the aqueous, undiluted GA solution (2.3E−3 mM) were used for the ASAP measurement, a maximum amount of approximately 4.6 pmol GA (0.78 ng) could be present. Using the standard method (Table A1, method 1) with 10 μA corona current (Exp. 1H) or a similar method (Exp. 3: 3 μA) for the measurement of aqueous GA solutions (Exp. 1H) at two concentrations (2.3E−3 mM and 100‐fold diluted (2.3E−5 mmol)), only an important peak at m/z 169, characteristic for GA, was observed (2.3E−3 mM: *I*(169) = 3E6 and 2.3E−5 mM: *I*(169) = 2E7). In contrast to TA ink, under these conditions, the intensities of signals in the region of m/z 300 to 304 were negligible at all retention times, and almost no EA signal at m/z 301 could be detected (< 1%). Furthermore, ASAP measurements were performed with GA powder. Since inks can only be measured as solids, a measurement of GA without water is better comparable with the measurement conditions used for inks. Because water is eliminated during condensation reactions, it might change the reaction pathways and influence the measurement result. Moreover, to improve the separation of peaks, the methods were prolonged to 6 min. To prevent the immediate decomposition of thermolabile species, a temperature ramp was used instead of a constant temperature of 650°C (as method 1). The parameters were varied to study their influences.

#### 3.5.2 Powder Measurements of GA

GA samples in the ng range, as obtained with solutions, could not be prepared by touching the dry GA powder with a wetted glass rod. Instead, this method resulted in quantities of approximately 100 μg. Using a collision energy of 6 eV and a sample cone voltage ≤ 30 V, the GA peak appeared in the chromatogram between 3 and 5.75 min, characterized by a predominant peak at m/z 169 in the mass spectra. The peak at m/z 169 was in part accompanied by a small peak at m/z 125, corresponding to an ionized pyrogallol species. Similar findings (Table 3) have also been reported elsewhere [9, 10, 33, 34]. In addition to that, at a collision energy of 6 eV equally high, significant signals at m/z 303 and 304 were obtained (Table A1: Exp. 4). With collision energies of 6 and 20 V (Table A1: Exp. 4 and Exp. 5), the signals corresponding to the ions of the EA isotopologues at m/z 301 and 302 remained small at higher temperatures (Table A1: Exp. 5). In both cases, the signals of the ions at m/z 303 to 305 exhibited a similar profile to that observed with TA (Figure 8), but the exact relative intensities varied (Figure 7).

**TABLE 3:** Signals observed in ASAP measurements of GA in the negative ionization mode and their interpretation, in the order of the monoisotopic ions (RA: radical anion) with their minor isotope signals and theoretical relative intensities (for experimental details, see the number of the experiment (Exp.) in Table A1 and Supporting Information: Section 4).

At m/z		Δm/z		Interpretation [26, 27, 28]	
Obs.	Calc.^a^	mDa	Assignment	Elemental composition	Exp.
69.0343	69.0340	0.3	97–28	C_4_H_5_O_1_: C_5_H_5_O_2_–CO	6
79.0180	79.0184	−0.4	97–18	C_5_H_3_O_1_: C_5_H_5_O_2_–H_2_O	6
81.0350	81.0345	0.5	125–44	C_5_H_5_O_1_: C_6_H_5_O_3_–CO_2_	6
97.0275	97.0290	−1.5	125–28	C_5_H_5_O_2_: C_6_H_5_O_3_–CO	6
107.0204	107.0119	8.5	125–18	C_6_H_3_O_2_: C_6_H_5_O_3_–H_2_O	6
125.0234	125.0239	−0.5	169–44	C_6_H_5_O_3_: C_7_H_5_O_5_–CO_2_	6
169.0142	169.0137	0.5	170 − 1p	C_7_H_5_O_5_: GA^−^ = GA–H^+^	5
170.0170	170.0176	−0.6	169 + 1n	C_7_H_6_O_5_: GA^−^: first isotopic peak (7.8%)	5
171.0190	171.0210	−2.0	169 + 2n	C_7_H_6_O_5_: GA^−^: second isotopic peak (1.3%)	5
172.0199	172.0243	−4.4	169 + 3n	C_7_H_6_O_5_: GA^−^: third isotopic peak (0.09%)	5
300.9984	300.9984	0.0	2 · 170–2 · 18 − 2 − 1p	C_14_H_5_O_8_: EA^−^ = 2 GA–2 H_2_O–H_2_–H^+^	7
302.0042	302.0023	1.9	301 + 1n	C_14_H_5_O_8_: EA^−^: first isotopic peak (15.5%)	7
303.0168	303.0057	11.1	301 + 2n	C_14_H_7_O_8_: EA^−^: second isotopic peak (2.8%)	7
304.0238	304.0091	14.7	301 + 3n	C_14_H_7_O_8_: EA^−^: third isotopic peak (0.3%)	7
303.0129	303.0141	−1.2	2 · 170–2 · 18 − p	C_14_H_7_O_8_: MP^−^: 2 GA–2 H_2_O–H^+^	4, 5
304.0206	304.0175	3.1	303 + 1n	C_14_H_7_O_8_: MP^−^: first isotopic peak (15.5%)	4, 5
305.0238	305.0208	3.0	303 + 2n	C_14_H_7_O_8_: MP^−^: second isotopic peak (2.8%)	4, 5
306.0234	306.0247	−1.3	303 + 3n	C_14_H_7_O_8_: MP: third isotopic peak (0.3%)	4, 5
304.0206	304.0219	−1.3	2 · 170–2 · 18 + e	C_14_H_7_O_8_: RA‐MP^−^: 2 GA–2 H_2_O	4, 5
305.0238	305.0258	−2.0	304 + 1n	C_14_H_7_O_8_: RA‐MP^−^: first isotopic peak (15.5%)	4, 5
306.0234	306.0292	−5.8	304 + 2n	C_14_H_7_O_8_: RA‐MP^−^: second isotopic peak (2.8%)	4, 5
307.0302	307.0325	−2.3	304 + 3n	C_14_H_7_O_8_: RA‐MP^−^: third isotopic peak (0.3%)	4, 5
321.0241	321.0247	−0.6	339–18	C_14_H_9_O_9_: 2 GA–H^+^–H_2_O	7, 8^b^
339.0349	339.0352	−0.3	2 · 170 − p	C_14_H_11_O_10_: 2 GA–H^+^	8

^a^ The given values are given as provided by MassLynx. They do not include the electron mass *m*_*e*_ (see Supporting Information: Section 1).

^b^ Very small signal at m/z 321.

**FIGURE 9:**
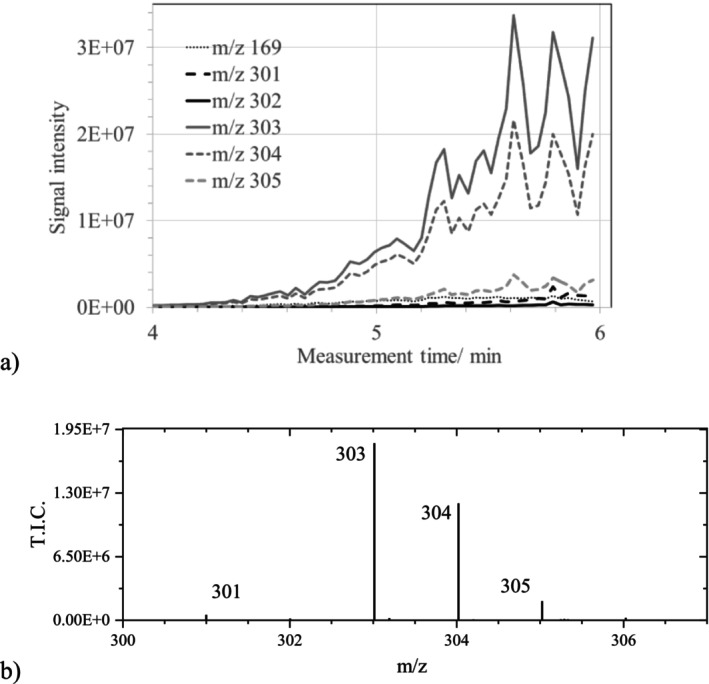
(a) Traces of the ion signals at m/z 301 to 305 obtained from the chromatogram of an ASAP measurement of GA powder in the negative ionization mode at a collision energy of 20 V (Table A1, Exp. 5). (b) The mass spectrum obtained at 5.8 min from the same chromatogram. The signal at m/z 304 is significantly higher than the isotope signal (~15.6%) belonging to m/z 303.

With the higher collision energy of 20 eV, the GA peak at m/z 169 became negligibly small throughout the entire chromatogram. Furthermore, several breakdown products of GA (e.g., m/z 151.005; see Table 3) and of the intermediate product (MP) with lower masses were formed. However, as shown in Figure 9, the signals for the ions at m/z 303 and 304 increased significantly, the signal at m/z 304 decreasing relative to that at m/z 303. There was no signal of the EA anion, the signal at 301 belonging to the background (see Section 3.3).

Using MassLynx, the composition C_14_H_8_O_6_ could be attributed to the ion m/z 304 with a fit confidence of 100%. With still higher collision energies (30 eV) and at a sample cone voltage of 120 V (Table A1, Exp. 6), in addition to the signals at m/z 169 and 125 [9, 10, 33, 34], signals of additional breakdown products were observed in the MSMS mode (Table 3). Because these are not relevant for the formation of EA, the corresponding mass spectra are given in Supporting Information: Section 3.3.

Using a moderate sample cone voltage of 60 V and a collision energy of 6 eV (Table A1, Exp. 7), in contrast to the other measurements, a considerable increase of EA at m/z 301 and 321, with small signals at m/z 304, was obtained (Table 3). The signal at m/z 169 was still present with about 75% of the intensity of that of EA. Although the m/z value matched well, the signals at m/z 321.0229 could not be unambiguously attributed with MassLynx to the most likely species C_14_H_9_O_9_ (m/z 321.0247) because the latter was only attributed with a fit confidence of 65.5%. The composition C_7_H_13_O_14_ with a higher oxygen content had a fit confidence of 32% due to a misassignment (Supporting Information: Section 1.3). This interference was explained by the other peaks that were observed near to the signals at m/z 321 (magnified section, Table A1, Exp. 7) but also near to the signal at m/z 339 in case of a collision energy of zero (see also Supporting Information: Section 4).

**FIGURE 10:**
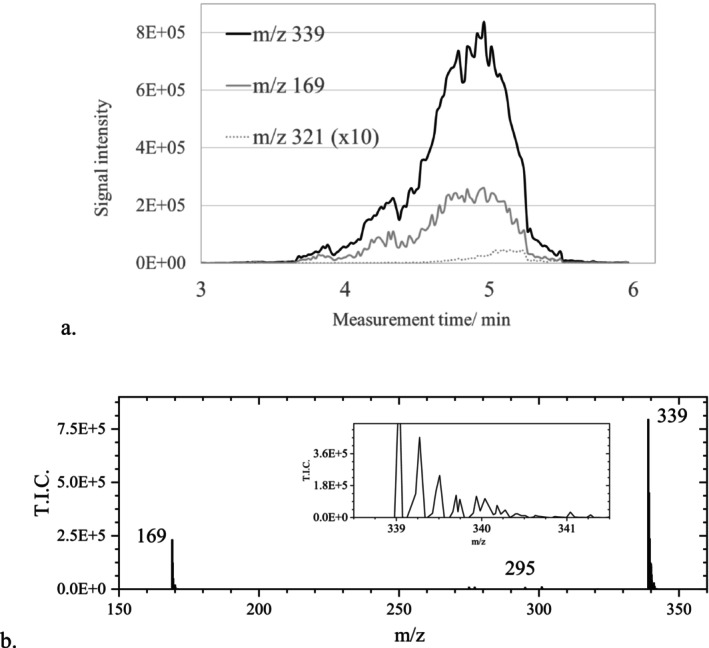
(a) Intensity courses of the ions at m/z 169, 321, and 339 observed in an ASAP measurement with GA in the negative mode with a collision energy of zero (more details see Table A1, Exp. 8). (b) Mass spectrum obtained at 4.9 min in the chromatogram.

Performing ASAP experiments with no collision energy, prominent signals were detected at m/z 169.0147 and at m/z 339.0347, about twice the mass of GA, accompanied by a small signal at m/z 321 (Figure 10b). As for m/z 321, additional peaks at non‐integer m/z were observed for the ion at m/z 339. However, despite such additional signals, the composition C_14_H_11_O_10_ could be attributed to the m/z value 339.0349 with a fit confidence of 98%.

Minor isotopic peaks appeared at m/z 340.0383, 341.0406, and 342, with slightly varying relative intensities of ~14.8% (calc. 15.6%), 3.7% (calc. 3.1%), and 0.43% (calc. 0.37%). Figure 10a shows clearly that the signal courses of m/z 321 and 339 are related to that of GA at m/z 169.

The signal at m/z 169.0142 could be assigned to GA with a fit confidence above 99.9% during the whole measurement time. Thus, measurements with a collision energy of zero favor the determination of GA with a higher fit confidence. The signals observed at m/z 339.2718, 339.5076, and 339.7014 exhibit similar decimal places as the signals near to the ion at m/z 321.0247. Such fractional numbers are typical for ions corresponding to those observed for multiply charged ions of higher molecule associates (see Supporting Information: Section 4).

In Table 3, a summary of the ions in the mass spectra of ASAP measurements of GA is given. According to Mass Lynx, the intermediate product MP was present as both a normal anion and a radical anion. The formation of EA from GA, also in the case of TA and inks, and the relation of the latter ions to the formation of EA will be discussed in Section 3.6.

In contrast to ink measurements (Table 2), the species at m/z 302 has not been observed in ASAP measurements of GA. With tannins, frequently signals of EA at m/z 301 with varying signal intensities were found. Whereas the composition of tannin was largely unknown (Supporting Information: Section 2), the presence of EA in GA could be excluded. Therefore, it can be concluded that the signal of EA at m/z 301 must have been formed from the utilized substances. Without additional reaction products, the intensities of the peaks at m/z 302 and 303 should correspond to those calculated for the minor isotopic peaks of the EA anion. However, the measured relative intensities were significantly higher than the expected theoretical relative intensities, 26% instead of 15.5% for the first signal and 8% instead of 2.8% for the second isotope signal. Therefore, the peak at m/z 304 cannot be only the third minor isotopic peak of the anion of EA (m/z 301), which would be much smaller (0.3%). Moreover, the intensive peaks at higher m/z values such as m/z 304 up to 306 or even 307 cannot be explained by minor isotopic peaks of the EA anion. So, there were other ions present at m/z 303 and 304. Because the negative mode was present, the ion at m/z 303 must be the anion of a molecule (MP^−^) of the molar mass *M* = 304 g/mol. Because the m/z value of MP is by two units higher (m/z + 2), it should have two hydrogen atoms more than EA, and it is hypothesized that this is an intermediate molecule or the precursor of EA. The minor isotopic peak at m/z 304 of the product anion at m/z 303 would exhibit a relative intensity of 15.5% compared with the signal at m/z 303. However, the observed signal intensity for the ion at m/z 304 was several times higher than the signal at m/z 303 itself. Thus, also the peak at m/z 304 cannot be merely the minor isotopic peak of the peak at m/z 303. An ion with approximately the same even value of m/z as the mass of the expected neutral product (*M* = 304 g/mol) can only be the radical ion of the precursor of EA. For the separation of the first minor ^13^C isotopic peak (m/z = 304.0175) of the normal MP anion at m/z 303 and the electron capture product (m/z = 304.0214) of the neutral molecule, a separation of *R* = 77 953 would be necessary. Because the resolution of the mass spectrometer was ~22 000, such peaks could not be separated. Neglecting the influence of the isotope signal of the ion at m/z 303, the composition C_14_H_8_O_6_ was attributed to m/z 304 by MassLynx with a fit confidence of > 90%, frequently > 98%, in various measurements with GA. However, due to the interference with the signal at m/z 304, the ion at m/z 303 could not be identified unambiguously with a satisfying fit confidence by MassLynx. Thus, the signals indicate that two species were present with ions at m/z 303 and 304. However, it was not possible to explain all observed intensities in the range 300 to 307 based on such ions and corresponding isotopic peaks (Supporting Information: Section l.5).

### 3.6 EA

**FIGURE 11:**
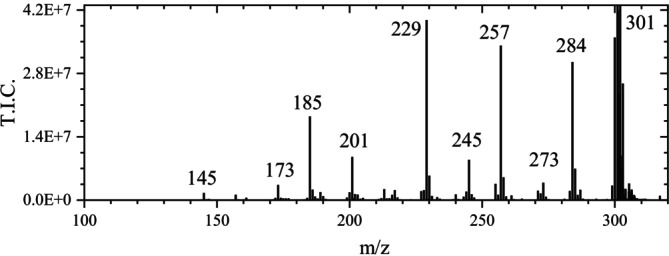
Mass spectrum of the decomposition products of ellagic acid (magnified) obtained from a chromatogram of the ASAP measurement of ellagic acid powder at 5.8 min measurement time in the negative ionization mode (Table A1, Exp. 5B) (base peak at m/z 301).

Except for the signal at m/z 602.9995 (2 M–H), reported by Fernandes et al. [35], all MS signals previously published by Fernandes et al. [35] (LC‐ESI‐HRMS/MS), Mämmelä et al. [9] (ESI), and Wyrepkowski et al. [10] were also observed in the ASAP measurements of this work in the negative mode (Figure 11).

Mämmelä et al. [9] reported signals at m/z 147.01, 157, 229, 257, 272.1, 283.99, and 300.998 (values cited as given by the author). They explained the species at m/z 272 by a neutral loss of 29 from m/z 301. However, because a mass loss of 29 (e.g., H‐CO^+^) would not represent a neutral loss and the signal at m/z 272, also observed in this work, was less intense than the already small signal at m/z 273, it is suggested that m/z 273 results from m/z 301 by a mass loss of 28 (CO) (see Table 4). Furthermore, the signal corresponding to m/z 147 [9] could not be confirmed. In contrast to the signal at m/z 145, the one at m/z 147 appeared negligibly small in the ASAP measurements in this work and could not be attributed to a chemical process (a loss of 26 (ethyne) appearing unlikely). Considering that Mämmelä et al. [9] reported signals at m/z 272 instead of m/z 273 (this work) and at m/z 147 instead of m/z 145 (this work), a calibration issue in the lower mass range or another way of fragmentation appears likely. Finally, the mass loss of 72, given by Mämmelä et al. [9] to explain m/z 229, was identified in this work as the loss of carbon dioxide and carbon monoxide. Feretti et al. [24] also reported signals at m/z 301, 284, 257, 229, 185, and 145 for mass spectra obtained by ESI‐Q‐ToF but without giving an interpretation.

**TABLE 4:** Signals observed with ASAP measurements of EA in the negative ionization mode. Considerably different data in literature are given in brackets (for experimental details, see the number of the experiment (Exp.) in Table A1).

At m/z		Δm/z		Interpretation [36, 37, 38], [35]	
Obs.	Calc.^a^	mDa	Assignment	Elemental composition of anions	Exp.
101.0382	101.0391	−0.8	145–44; 129–28	C_8_H_5_: C_9_H_5_O_2_–CO_2_; C_9_H_5_O–CO	5B
117.0330	117.0340	7.0	145–28	C_8_H_5_O_1_: C_9_H_5_O_2_–CO	5B
129.0347	129.0340	0.7	157–28	C_9_H_5_O_1_: C_10_H_5_O_2_–CO	5B
145.0280	145.0290	−1.0	173–28	C_9_H_5_O_2_: C_10_H_5_O_3_–CO	5B
265.9845	265.9851	−0.6	284–18	C_14_H_2_O_5:_ C_14_H_4_O_7_–H_2_O	5B
157.0306	157.0290	1.6	185–28 or 201–44	C_10_H_5_O_2_: C_11_H_5_O_3_–CO or C_11_H_5_O_4_–CO_2_	5B
173.0229	173.0239	−1.0	201–28	C_10_H_5_O_3_: M^−^–CO_2_–3 CO	5B
185.0229	185.0239	−1.0	229–44	C_11_H_5_O_3_: M^−^–CO_2_–CO–CO_2_	5B
			213–28	M^−^–CO_2_–CO_2_–CO	
201.0190	201.0188	0.2	229–28	C_11_H_5_O_4_: M^−^–CO_2_–2 CO	5B
			245–44	: M^−^–2 CO–CO_2_	
229.0144	229.0137	0.7	257–28	C_12_H_5_O_5_: M^−^–CO_2_–CO	5B
240.0068	240.0059	0.9	284–44	C_13_H_4_O_5_: C_14_H_4_O_7_–CO_2_	5B
245.0107	245.0086	2.1	301–2*28?	C_12_H_5_O_6_: M^−^–2 CO (3.39 Fit conf %) *	5B
	245.0450	−34.3		C_13_H_10_O_5_ (94.75% Fit conf. %)^b^	
255.9998	256.0008	−1.0	284–28	C_13_H_4_O_6_: C_14_H_4_O_7_–CO	5B
257.0086	257.0086	0.0	301–44	C_13_H_5_O_6_: M^−^–2 CO_2_	5B
273.0050	273.0035	1.5	301–28	C_13_H_5_O_7_: C_14_H_5_O_8_—CO	5B
282.9913	282.9879	3.4	301–18	C_14_H_3_O_7_: EA^−^–H_2_O	5B
283.9956	283.9957	−0.1	302–18	C_14_H_4_O_7_: RA‐284: RA‐EA‐H_2_O	5B
299.9989	299.9906	8.3	302–2 + e	C_14_H_4_O_8_: RA‐300: C_14_H_5_O_8_: M–H_2_ + e^−^	5B
300.9984	300.9985	−0.5	M^−^ = 302‐ p	EA^−^ C_14_H_5_O_8_: M–H^+^ [9, 35]	5B
302.0030	302.0023	0.7	301 + 1n	EA^−^: first isotopic peak (15.5%)	5B
303.0040	303.0057	−1.7	301 + 2n	EA^−^: second isotopic peak (2.8%)	5B
302.0030	302.0068	−3.8	302 + 1e	C_14_H_6_O_8_: RA‐EA: 2 GA–2 H_2_O–H_2_	5B
303.0040	303.0102	−6.2	302e + 1n	RA‐EA: first isotopic peak (15.5%)	5B
304.0060	304.0135	−7.5	302e + 2n	RA‐EA: second isotopic peak (2.8%)	5B

^a^ The given values are provided by MassLynx. They do not include the electron mass m_*e*_.

^b^ Supporting Information Section 1.4.

At the beginning of the EA peak in the chromatogram (at 4.7 min) of the ASAP measurement, the signal at m/z 300.9980 of EA was attributed by MassLynx to EA (m/z 300.9984) with a fit confidence of 100%, despite the presence of background peaks. Correspondingly, the isotopic peaks had relative experimental signal intensities close to the calculated values—m/z 302: 15.93% (calculated 15.48%), m/z 303: 3.02% (calculated 2.78%), and m/z 304: 0.20% (calculated 0.30%). However, at the end of the measurement (5.9 min)—probably related to the increased temperature—the peak at m/z 300.9984 was slightly shifted to m/z 301.003 and was attributed to C_14_H_5_O_8_ (EA) by MassLynx with only 64% fit confidence (and (due to a misassignment) to C_7_H_9_O_13_ with a fit confidence of 36%), indicating interference of the signals at m/z 302 and 303 (Supporting Information: Sections 1.4 and 1.5).

The observed time‐dependent variation of the fit confidence for EA in the ASAP measurement is likely due to the formation of species at m/z 300 and m/z 302. In both cases, the resulting isotope signals interfere with those of EA.

The even ion at m/z 299.99 in the mass spectrum of EA was also observed in the case of TA ink. Because a high signal intensity of the radical anion at m/z 299.99 should influence the minor isotopic peaks at 301 and 302, it must be considered for measurements including the ion at m/z 301. Because there were no peaks corresponding to the minor isotopic peaks at their precise m/z values of m/z 300.9945 and 301.9979 (only at m/z 300.9330 and 301.0030 as well as 302.0030 and 302.0530), it is concluded that the minor isotopic peaks of m/z 300 at m/z 301 and 302, needing a resolution of 77 000 to be separated from the base peak at m/z 301 and the peak at 302 (radical anion), are merged with the peaks of the species at m/z 301 (EA anion). As in the case of the sample TA ink 1, the signal intensity at m/z 302 amounts to 50% of the base peak at m/z 301, which largely exceeds the intensity of a first minor isotopic peak. Due to its even m/z value, which reveals a species with an odd number of electrons, the signal at m/z 302.0063 must be due to the radical cation of the composition C_14_H_8_O_6_, which is merged with the minor isotopic peak of EA, because a resolution of *R* > 377 000 would be necessary for the resolution. The presence of the radical anion at m/z 302 also explains increased intensities at m/z 303 and 304, which should have much lower intensities of ~2.8% and ~1% relative to the peak at m/z 301, if they were merely the second and third minor isotopic peaks of the latter. Correspondingly, the ratio of the intensities at m/z 303 (*I*_0_) and 304 (*I*_1_) has been bigger than expected, frequently being *I*_1_/*I*_0_ ≥ 4%, thus differing from the theoretical value of 2.8%. There is only one peak at m/z 303.0022, and it was concluded that the first minor isotopic peak at m/z 303.0096 of the radical anion (m/z 302) is merged with the second minor isotopic peak (at m/z 303.0052) of the deprotonated molecule (at m/z 301). Because the MS had a resolution *R* of 22 000 and the peaks at 303.0052 and 303.0096 would need a resolution of *R* > 68 000, the latter could not be resolved.

Although the ionic mass observed at m/z 283.996 is similar to that of the expected precursor ion for the decomposition according to “Elemental composition,” its formation is remarkable because it must be a radical anion to explain its observability. Fernandes et al. [35], who reported species at m/z 301, 185, 229, and 284, interpreted the species m/z 283.996 by the loss of a hydroxyl radical from EA and did not further consider it. However, the formation and detection of radical anions under ESI and ASAP conditions have been rarely reported yet [39]. On the one hand, the loss of radicals (such as a hydroxyl radical) from even electron species, such as an EA molecule or anion (as suggested by Fernandes et al. [35]), is generally considered forbidden [31]. On the other hand, radical losses are well known to occur for hydrocarbons and peroxides at increased temperatures. Because a temperature of 650°C was applied in ASAP measurements, such a violation of the rule cannot be excluded. However, in both cases—peroxides and hydrocarbons—covalent bonds with a lower dissociation energy are broken, which are not present in the case of the EA molecule, and make it highly improbable that a hydroxyl radical is lost from the EA molecule. However, an alternative explanation would be the loss of a neutral group from a radical cation at m/z 302 as observed in this work. The latter could be formed by electrochemical oxidation or under the influence of oxidizing iron, as in the case of inks. Thus, a reasonable explanation for the formation of a radical anion at m/z 284 is a water loss from the radical anion at m/z 302. Furthermore, there was also a smaller signal at m/z 282.99, generally being significantly smaller than that at m/z 283.99, which could indicate that water loss might also occur to a small extent from the anion at m/z 301. An ion at m/z 257 can be explained by the loss of carbon dioxide. However, due to the present ester structure, this loss should be much more difficult than in the case of free carboxylic acids. Although the m/z difference of 56 can only be explained by a neutral loss of two carbon monoxide molecules, the corresponding, chemically most likely, composition C_12_H_5_O_6_^−^ could be attributed to the ion at m/z 245 only with a fit confidence of 3.4%, which should be due to the small signal intensity and significant interference of the background signals. The suggested composition C_13_H_9_O_5_, attributed to m/z 245 by MassLynx with a fit confidence of 94.75%, could not be explained by neutral losses of EA (see Supporting Information: Section 1.3).

## 4 Summary of Results and Discussion of the Formation of EA

### 4.1 Literature Results

Whereas the synthesis of EA from GA derivates is quite challenging (Supporting Information: Section 6), it is known that EA is formed by autoxidation in the frame of the aging process of inks [24]. Whereas it cannot be ruled out that the electrochemical and thermal conditions in ESI measurements lead to oxidation and coupling of analytes [23], Feretti et al. [24] did not report the formation of EA, analyzing the extracts of fresh mock ink samples by HPLC‐ESI Q‐tof, which confirms the findings by own ESI measurements (Supporting Information: Section 2). However, for ASAP measurements, the formation of dibromo benzenes and phenols was reported to result in coupling of similar analytes to form diphenyl ethers and bromo diphenyl compounds [39]. In addition to diphenyl ethers, the formation of biphenyls (and phenoxybiphenyls and triphenyls) from bromobenzenes and phenols was also observed [39]. The species found in ASAP measurements of bromophenols indicate that, in principle, in addition to the formation of EA, other reaction pathways are possible with the corresponding species. It is interesting to note that, in contrast to the coupling products of the bromo compounds, no other side products were produced from GA by the ASAP method if reaction conditions were adequate. Considering the structure of bromo diphenyls, being similar to that of EA and the postulated reaction products, the formation of the thermodynamically stable EA in the context of our experiments is plausible.

### 4.2 Formation of the Observed Species

#### 4.2.1 Molecule Adducts

In the frame of the ASAP measurement, an important condition for the reaction of two GA molecules is a sufficient concentration, which explains the presence of dimers in the case of powder measurements due to the higher sample amounts. At low collision energies (C.E. = 0), molecule adducts are formed, as they are also known for acetic acid in the vapor phase [40], which cause signals at m/z 339.

**FIGURE 12:**
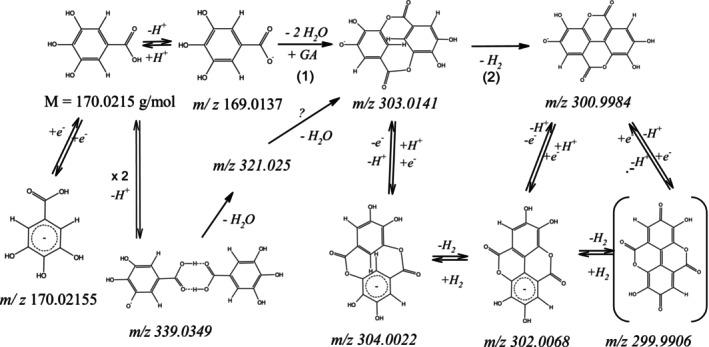
Formation of EA in the negative mode and identified free radical anions.

At higher temperatures and sample cone voltages, such dimeric adducts can lose water, resulting in an ion at m/z 321. Because the ion at m/z 339 can lose water in several ways, the structure of the ion m/z 321 is unknown. However, with regard to the precursor at m/z 339, the ion at m/z 321 could be an intermediate in the formation of the anion at m/z 303 (Figure 12). Because one molecule of water is eliminated, a covalent CO‐O‐ bond to the second molecule is possible. Because the signal course of m/z 321 followed that of GA at m/z 169 and that of the dimeric species at m/z 339, the ion is likely to be formed by water loss of the latter and might be the precursor of the species MP^−^ (m/z 303).

#### 4.2.2 Covalent Dimers

The main reaction products, identified by ASAP measurements in the negative ionization mode, had signals at the m/z values 304, 303, and 301, where m/z 301 was proved to be due to EA in Section 3.6 by the analysis of its fragments and comparison with literature. Furthermore, the intensities of the signals at 303 and 304 exceeded in several cases largely those of the isotopic peaks of the peak of EA at m/z 301. Thus, the signals at m/z 303 and 304 cannot only consist of the isotopic peaks but should correspond to other species, which are similar to EA but are two (C_14_H_7_O_6_) and three m/z units bigger. Although at this place their composition is not exactly clear, this should be due to a corresponding higher number of hydrogen atoms (C_14_H_8_O_6_) relative to the EA anion (C_14_H_5_O_6_), because they must be related to the thermal condensation of two GA molecules, involving the formation of two C‐O bonds, without oxidation to EA. This is shown in Figure 12 where the resulting product (MP) is a cyclic compound featuring two linkages (each of length two) in the meta position of the benzene rings, which places it in the class of [2,2]‐metacyclophanes [41, 42, 43]. As with the formation of other cyclophanes, this reaction is favored by gas phase conditions and small concentrations [44], relative to normal laboratory conditions, albeit relatively high concentrations compared with typical MS ionization conditions. These findings, suggesting the formation of the normal MP anion at m/z 303 (step 1, Figure 12) by the condensation of GA molecules under ASAP conditions, are discussed in the following with respect to the results obtained with samples of inks, GA and TA (see also Table 5). In the case of measurements of diluted GA solutions, only the signal at m/z 169 was observed, but for solid GA mainly peaks in the range 303 to 307 appeared. The appearance of reaction products at m/z 303 in the latter case can likely be attributed to the higher quantity of GA and the absence of water that favors condensation products (MP). In solution, however, the presence of water and the lower concentration prevented the condensation of the molecules (Figure 12, step 1). A similar explanation may apply to the GA ink, which contained only a small proportion of GA. This interpretation is supported by the fact that only negligible traces of condensation products were observed under ESI conditions either. The ASAP measurements of TA samples resulted in a significant signal at m/z 169 in the case of solid TA powder or its undiluted solution of 2.3 mM/L and, as also observed for GA powder, in predominant peaks in the region of m/z 300 to 304, the signal at m/z 303 corresponding to the intermediate product (MP). In contrast to EA, a substance related to m/z 304 has not been reported yet as a natural component of TA. Thus, it is not a component of TA and indicates that a reaction occurred. An important difference between TA ink and GA ink is the presence of a glucose unit as a part of the TA molecule. However, while glucose decomposition may generally elevate the radical species levels, the precise mechanism by which it promotes the formation of the ions at m/z 303 and 304 remains unclear. The origin of species with even masses, such as m/z 304, is discussed at the end of this section.

**TABLE 5:** Overview of the results obtained in this work.

	TA					GA		
	ESI	ASAP				ASAP		
m/z	Solution		Pure solid	Ink1	Ink2	Solution	Pure solid	GA ink
169	s	s	m	—	—	s	w	vs
300	—	w	w	vw	s	—		—
301	—	—	m	vs	s	—	m	—
302	vw	—	w	s	vs	—	w	—
303	—	vs	s	w	vs	—	vs	—
304	—	vs	vs	vw	w	—	vs	—
305	—	s	m	—	w	—	m	—
306	—	w	w	—	w	—		—
>500	7–9 signals (s)^a^	No sample signals						

*Note:* m, mean; s, strong; vs, very strong; vw, very weak; W, weak.

^a^ See Figure 6.

In case of the studied TA ink 2, the signal at m/z 303 also was predominant, but accompanied by an important signal of the EA anion at m/z 301, which was the most prominent signal in case of TA ink 1, whereas the signal at m/z 169 was absent. In contrast to TA and GA solutions, the dehydrogenation (oxidation) of the product m/z 303 under dry conditions and at high temperatures was favored in the case of inks due to the presence of iron III ions. Because nonvolatile and very polar substances cannot entirely vaporize during the ASAP measurement [18], the ink decomposes and the polyphenol compounds are oxidized due to the higher oxidation state of the iron III ion, which is also known to oxidize paper [45]. The loss of hydrogen of MP (Figure 12, step 2) via subsequent oxidation (Figure 12, step 2) to the EA anion at m/z 301 might also be favored by the steric interaction of the two hydrogen bonds in the molecule. The different signal intensities in the range m/z 301 to 305 of the two ink samples of the same ink using the same method (Table A1, method 1) indicate different oxidation states of the ink samples due to a varying conversion with oxygen. Because only atmospheric oxygen was used for the oxidation of the ink, as this is generally done for mock inks [46], it is likely that the particles of the second sample were less exposed to air than those of the first (TA ink 1). This could originate from a slight variation in sampling—if, for example, the particles of the sample TA ink 1 were taken from nearer the surface of the ink batch than the second. In contrast to historical inks, which had hundreds of years to oxidize via oxygen diffusion, recently fabricated mock samples cannot be expected to have a homogeneous composition. The lower oxidation state of the second ink sample also led to important signals at m/z 300 and at m/z 302, which will be discussed at the end of the section. At a higher sample cone voltage, a collision energy of 6 eV, and elevated temperatures, also, ASAP experiments with GA resulted in the formation of the ion at m/z 301 (MP^−^). Thus, in this case, the oxidation is due to the elevated sample cone voltage instead of the iron ion of the ink.

The results with Arabic gum containing gum might indicate that Arabic gum can reduce the effect of the higher temperature and stabilize the GA signal, probably due to a reducing effect.

#### 4.2.3 Radical Anions

The intensity deviations of the first minor isotopic peak of GA during the ASAP measurement and the corresponding low fit confidence for GA can be best explained by a radical anion at m/z 170 interfering with the minor isotopic peaks of GA.

Similarly, the signals at m/z 300, 302, and 304 with odd electron numbers and even masses hampered in part the analysis of the EA ion and other species. The observation of such radical anions under the ASAP conditions can be explained by the generally enhanced stability of free radicals at higher temperatures. On the other hand, the formation of radical anions such as m/z 304 requires reductive conditions. Thus, to obtain this species, at least a deficiency of oxygen relative to the amount necessary for the full oxidation of the sample is necessary.

Among the radical anions, the species at m/z 304 were the most prominent. As explained earlier, MP (*M* = 304 g/mol) is an intermediate in the reaction from GA to EA but could also form from the anion m/z 303. Thus, the negatively charged radical ion m/z 304 was formed in the case of ink sample 2 from MP by uptake of an electron but not in the case of ink sample 1. This can also be explained by the incomplete oxidation of ink sample 2 compared with ink sample 1. The stability of the radical anion m/z 304 can be understood by two factors. First, the compound has two benzene rings, which can accept an electron and are influenced by the negative mesomeric effect of the carboxyl group, stabilizing the negative charge. Secondly, due to the reductive conditions, an excess of electrons is available for such a reaction.

The m/z value of 302 corresponds to the molar mass of the neutral EA molecule. However, being neutral, the latter could not be detected by the MS. The ion at m/z 302 can be explained by the loss of hydrogen from the species at m/z 304 (Figure 12) due to the iron ion of the ink or by reduction of EA via electron transfer during the ASAP experiment. A further dehydrogenation can lead to the radical species at m/z 300, as also observed with TA ink 2. The high intensity at m/z 305 could be explained theoretically by the anion of another species with *M* = 306 g/mol, enhancing the signal height compared with the given minor isotopic peaks, but this is difficult to rationalize by a corresponding structure. Whereas the results of the ASAP measurement for solid TA and GA were similar, the formation of the intermediate MP in the case of the TA solution, but not with the GA solution, is not clear.

The even ion at m/z 299.99 that was also observed in the case of TA ink, must be a radical anion (see also Table 2) stemming from the fragmentation of the EA anion. Its tentative structure should be similar to that of quinones, which are quite stable compared with easily oxidizable hydroquinone (compare quinone‐hydroquinone electrode [47]) and polyphenol compounds such as EA and GA. A similar species, the radical anion of tetrabromoquinone, was reported by Skopalová et al. [39].

## 5 Conclusions and Outlook

The target of this work was to prove the occurrence of reactions in ASAP measurements of GA inks, tannin, and GA and identify intermediate products in the negative ionization mode. The measurements revealed the formation of several reaction products, which in part depend on the collision energy. At a collision energy of zero, molecule associates of GA are formed (m/z 339). Using the standard collision energy of 6 eV, ASAP measurements with solid GA and tannin gave EA and/or a [2] metacyclophane via thermal condensation and cyclization. In the case of ASAP measurements, especially with inks, a subsequent dehydrogenation of that intermediate led to the formation of EA, which appears to be favored by the iron III ion. This occurred even though the samples mainly contained gallotannins and GA, as verified by ESI. The precursor of EA, [2] metacyclophane, exhibited intensive signals at m/z 303 and 304 with additional peaks at m/z 305 and 306. Especially in the case of inks, important signals were identified as radical anions of [2] metacyclophane (m/z 304), EA (m/z 302), and an oxidation product (m/z 300). It was shown that samples of the same mock ink can give very different ASAP measurement results, which could be interpreted by different oxidation states of different particles of the ink. This should be due to the formulation of the ink that does not permit obtaining a defined oxidation state because the oxygen of the air was used. On the one hand, to take account of the short time of oxidation, compared with historical inks, the standard procedure for mock samples should involve adding an oxidizing agent after the complexation of the iron by the tannin component if reproducible measurement results are desired. On the other hand, these results indicate that, unless the ASAP method is significantly modified and optimized concerning all parameters (gas flows, temperature, voltages), determining the original composition of iron‐gall inks from solid samples using ASAP is unlikely. A design of experiments (DOE) approach, specialized on the minimization (to optimize in further works the measurement method for inks), might help to better understand the system.

## Supporting information




**Figure S1:** Ion mass of gallic acid measured and calculated by MassLynx 4.2.2.
**Figure S2:** Peaks in the region 168.5 to 169.5. In the range 168.985 (169.01–0.025) to 169.035 (169.01 + 0.025), there are no other peaks.
**Figure S3:** HPLC chromatogram of a TA sample [11, 12].
**Figure S4:** Mass spectrum obtained from an ESI measurement of TA solution (2.3E−5 mmol) in the negative ionization mode (Table S7, Exp. 9), with magnification of the highest ion masses, showing the typical isotopic pattern of the signals.
**Figure S5:** Mass spectrum obtained at 5.294 min from the chromatogram of the ASAP measurement of GA in the negative mode (Table S7, Method 6).
**Figure S6:** Mass spectrum obtained at 5.920 min from the chromatogram of the ASAP measurement (MSMS mode) of GA in the negative ionization mode (Table S7, Method 6).
**Figure S7:** Traces of the ion signals at m/z 301 to 305 obtained from the chromatogram of an ASAP measurement of GA powder in the negative ionization mode (Table S6, Exp. 7).
**Figure S8:** Intensity courses of the ratios of the intensities of the signals at m/z 302 and 303 to that of 301 obtained from an ASAP measurement of EA in the negative ionization mode (Table S7, Method 9).

## Data Availability

The data that support the findings of this study are available from the corresponding author upon reasonable request.

## Appendix A

**TABLE A1:** Parameters of MS experiments in the negative ionization mode (R: 22k, sensitivity mode) Ref: DER setting: 99.9. A ramp is indicated by a second value in parentheses; for example, 50(650) corresponds to a ramp of 50°C up to 650°C.

Experiment		Mode		Gas flow		Electric parameter in parameter file			Temperatures (source offset)			Flow	Mass range
ASAP experiments													
	Exp. Nr.	MS	DR	Cone	Desolvation gas	Sample cone voltage	Corona current (or voltage)	Collision energy	Duration	*T*_sample_	*T*_source_	*v*_Infusion_	
—	—	—	—	L/h	L/h	V	μA/(kV)	eV/V	min	°C		μL/min	
TA and inks	1A) GA ink1 1B) Ink w/o Fe III 1C) TA ink 1 1D) TA ink 2 1E) TA 5x dil. 1F) TA. 10× dil. 1G) TA + Arab. Gum 1H) GA 100× dil.	MS	N	50	600	30	10 μA	6	3	650	120	5	50–1200
	2	MS^2^	N	50	600	30	10 μA	35	3	650	120	5	
GA	3	MS	N	50	600	30	3 μA	6	3	650	120	5	
	4	MS	ED	50	600	40	2 kV	6	6	50 (650)	100	5	50–2000
	5	MS	ED	50	600	40	2 kV	20	6	50 (650)	100	5	
	6	MS^2^	N	0	300	120	3 μA	30	6	50 (650)	120	5	
	7	MS	N	50	600	60	10 μA	6	6	50 (650)	120	5	
	8	MS	N	50	600	30	3 μA	0	6	50 (650)	120	5	
EA	5B EA	MS	ED	50	600	40	2 kV	20	6	50 (650)	100	5	

ESI experiments													
	Experiment	MS	DR	Cone gas	Desolvation gas flow	Sample cone voltage	Capillary voltage	C.E.	Duration	Desolvation temperature	*T*_source_	*v*_Infusion_	Mass range
	9	MS	N	0	300	150	2.5 kV	15	2	350	120	5	50–2000
