# Supplementary material for: Beware the Artifact: Generation of Ellagic Acid and Related Species During Atmospheric Solid Analysis Probe (ASAP) Measurements of Iron Gall Inks and Tannins in the Negative Ionization Mode
Source: J Mass Spectrom. 2026 Mar 25;61(4):e70045. doi: 10.1002/jms.70045 (PMC13017080; doi:10.1002/jms.70045)
Supplement: Supplementary file 1 — Figure S1: Ion mass of gallic acid measured and calculated by MassLynx 4.2.2. Figure S2: Peaks in the region 168.5 to 169.5. In the range 168.985 (169.01–0.025) to 169.035 (169.01 + 0.025), there are no other peaks. Figure S3: HPLC chromatogram of a TA sample [11, 12]. Figure S4: Mass spectrum obtained from an ESI measurement of TA solution (2.3E−5 mmol) in the negative ionization mode (Table S7, Exp. 9), with magnification of the highest ion masses, showing the typical isotopic pattern of the signals. Figure S5: Mass spectrum obtained at 5.294 min from the chromatogram of the ASAP measurement of GA in the negative mode (Table S7, Method 6). Figure S6: Mass spectrum obtained at 5.920 min from the chromatogram of the ASAP measurement (MSMS mode) of GA in the negative ionization mode (Table S7, Method 6). Figure S7: Traces of the ion signals at m/z 301 to 305 obtained from the chromatogram of an ASAP measurement of GA powder in the negative ionization mode (Table S6, Exp. 7). Figure S8: Intensity courses of the ratios of the intensities of the signals at m/z 302 and 303 to that of 301 obtained from an ASAP measurement of EA in the negative ionization mode (Table S7, Method 9). [file JMS-61-e70045-s001.docx]

***Supporting Information***

**Beware the Artifact: Generation of ellagic acid and related species during Atmospheric Solid Analysis Probe (ASAP) measurements of iron gall Inks and tannins in the negative ionization section 1.4mode.**

Thomas Willms

Bundesanstalt für Materialforschung und -prüfung (BAM), Unter den Eichen 44, 12205 Berlin, Germany

E-mail: [thomas.willms@bam.de](mailto:thomas.willms@bam.de) , Tel.: +49-40-42838-8773.

***Supplementary Figures***

[Figure S1: Ion mass of gallic acid measured and calculated by MassLynx 4.2.2 3](#_Toc222980853)

[Figure S 2: Peaks in the region 168.5 to 169.5. In the range 168.985 (169.01 - 0.025) to 169.035 (169.01 + 0.025), there are no other peaks. 5](#_Toc222980854)

[Figure S 3: HPLC chromatogram of a TA sample [11, 12] 8](#_Toc222980855)

[Figure S4: Mass spectrum obtained from an ESI-measurement of TA solution (2.3E-5 mmol) in the negative ionization mode Table7, Exp. 9), with magnification of the highest ion masses, showing the typical isotopic pattern of the signals. 8](#_Toc222980856)

[Figure S5: Mass spectrum obtained at 5.294 min from the chromatogram of the ASAP measurement of GA in the negative mode (in main article Table7, method 6). 10](#_Toc222980857)

[Figure S6: Mass spectrum obtained at 5.920 min from the chromatogram of the ASAP measurement (MSMS mode) of GA in the negative ionisation mode (in main article Table7, method 6). 10](#_Toc222980858)

[Figure S7: Traces of the ion signals at m/z 301 to 305 obtained from the chromatogram of an ASAP measurement of GA powder in the negative ionization mode (main article Table 6, Exp. 7) . 11](#_Toc222980859)

[Figure S8: Intensity courses of the ratios of the intensities of the signals at m/z 302 and 303 to that of 301 obtained from an ASAP measurement of EA in the negative ionisation mode (Table 7, method 9). 13](#_Toc222980860)

***Supplementary Tables***

[Table S 1: Masses of particles (in u) and calculated m/z values for EA and GA anions. 2](#_Toc222980861)

[Table S 2: Comparison of isotope mass with the sum of the most abundant element and a neutron. 3](#_Toc222980862)

[Table S 3: Isotopic abundancies of carbon, oxygen and carbon 4](#_Toc222980863)

[Table S 4: Intensity ratios for the isotope peaks of GA and EA. 5](#_Toc222980864)

[Table S 5: Signal intensities of the species at 303 to 305 with their corresponding minor isotope peaks (i.p.) in the ASAP measurement of the sample TA ink 2. 7](#_Toc222980865)

[Table S 6: Ions of the charge envelope at 321.023 11](#_Toc222980866)

[Table S 7: Ions of the charge envelope at 339.034 12](#_Toc222980867)

## Generalities

## Remarks concerning the MassLynx “Elemental composition tool”

All mass spectra have been acquired and evaluated using MassLynx 4.2. For the interpretation of m/z differences in mass spectra, the nominal mass was used (e. g. for the proton mass 1.007825 u


[7] simply 1 is given) although for calculations the exact mono­isotopic m/z values were taken. To distinguish radi­cal ions from neutral molecules, the dif­fe­rence in elec­trons was given by formally adding or sub­trac­ting the symbol e. Correspondingly, in view of the high resolution and precision of the mass spectro­meter, following the recommendations in literature


[7], for all own calculations of m/z values, the elec­tron mass was considered with a value of m_e_ = 5.48E-4 u. It should be noted, that the tool “Elemental composition” in MassLynx 4.2 produced erroneous m/z values for the measured anions by neglecting the negative charge (Table S 1).

Table S 1: Masses of particles (in u) and calculated m/z values for EA and GA anions.

| Particle contribution | Anion of GA | Anion of EA |
| --- | --- | --- |
| Mass contribution/ g | C_7_H_5_O_5_ | C_14_H_5_O_8_ |
| of C | 84.000000 | 168.000000 |
| of H | 5.0391255 | 5.0391255 |
| of O | 79.97458 | 127.9593 |
| Molar mass (neutral radical)/ g/mol | **169.013700** | **300.998450** |
| Mass of electron e^.^ | 5.485799090E-4 | 5.485799090E-4 |
| m/z of anion: | **169.014249** | **300.998994** |
| **m/z given by MassLynx** | 169.0137 | 300.9985 |
| Atomic masses                                     [7]/ u: C:12.000000 H: 1.007825; O: 15.994915; electron mass                                      [7]/ u: 5.48579909E-4 | | |
|  | | |

As an ex­ample, Figure S1 shows the deter­mination of the GA anion by the “Elemental composition” tool (screen shot) with the corres­pon­ding measured (m/z 169.0142) and calculated m/z values (m/z 169.0137). In this case, the m/z differ­ence of 0.3 mDa was primarily attributable to the electron mass missing in the calculation rather than to the measurement accuracy of the mass spectrometer.


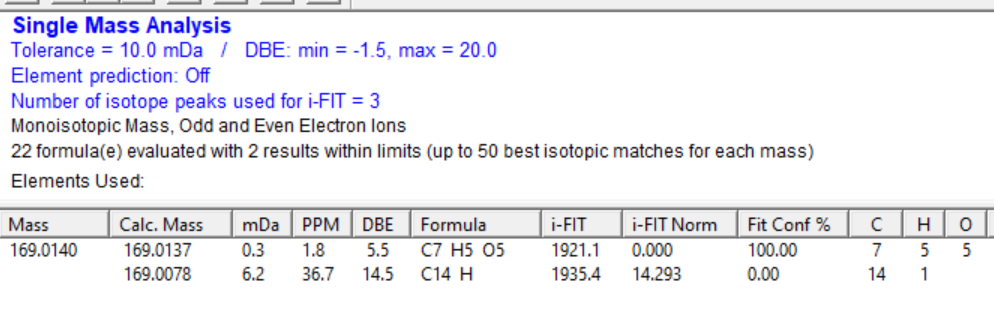


Figure S1: Ion mass of gallic acid measured and calculated by MassLynx 4.2.2

Due to this error, the m/z values, calculated by MassLynx and provided in the tables of the main ar­ticle, generally cor­respond to [m/z − e−]. If applicable, this has been indicated in the table notes accor­dingly. The data calculated by Mass Lynx were given, al­though they are **subject to a systematic er­ror** because they were the refe­rence used by Mass­Lynx for the determination of the species and compared with the measured values.

The error of the measurement was set to maximum of 10 mDa, to include species with larger devi­ations from the theoretical mass. To reduce further the possible results, the number of double-bond-equi­va­lents (DBE) (eq. 1) of the possible anions was limited to 20 (EA: 12 DBE, GA: 4 DBE).

| $DBE=N\left( C \right)+\frac{N\left( N \right)}{2}+1-\frac{N\left( H \right)}{2}-\frac{N\left( X \right)}{2}+\frac{N(q)}{2}$  N: Number of atoms; X: halogen atoms (oxygen has no influence), N(q): Number of negative charges | eq. 1 |
| --- | --- |

Generally, the com­po­si­ti­ons given by “Elemental composition” were analyzed concerning the com­positions, which had a number of car­bon, hydrogen and oxygen atoms, respectively, smaller or equal of that of two gallic acid mole­cules. Due to the relatively small masses and intensities of the con­sidered com­pounds in this work, in ge­neral, mostly the first and the second satellite peak were relevant for the evaluations, in some cases also the third. To make clear the difference in neutrons, the lat­ter is desig­nated in this work by for­mal­ly adding **+n** to the monoisotopic formula. Due to the mass effect, the mass of the isotopes is not the sum of the masses of a neutron and the most abundant atom (see Table S 2). Therefore, when in the tables of the main document formally a neutron n has been added to a formula, mathematically the difference of 1.00336 u between ^13^C and ^12^C atoms was added to the mono­isotopic ion mass to calculate the m/z value of the first ^13^C isotopologue, ignoring the isotopes of hydrogen and oxygen which have lower abundancies (Table S 3).

Table S 2: Comparison of isotope mass with the sum of the most abundant element and a neutron.

| Element E | | Hydrogen H | Carbon C | Oxygen O |
| --- | --- | --- | --- | --- |
| m(E)/ u | | m(^1^H) = 1.007825 | m(^12^C) =12.000000 | m(^16^O) =15.994915 |
| m(n) / u | | 1.008649 | | |
| m(E) + m(n) / u | | 2.016490 | 13.008665 | 17.003580 |
| Isotope mass/ u | | m(^2^H) = 2.014101 | m(^13^C) = 13.003355 | m(^17^O) = 16.999132 |
| difference | | 1.004217 | 1.003355 | 1.006277 |
| mass effect/ % | | 0.11856 | 0.04083 | 0.02617 |
|  |  |  |  |  |

The recommendation in lite­ra­ture


[7], to give four decimals for m/z up to 500, was followed in the tables. For clarity reasons, in the tables, the monoisotopic signals are grouped together with their isotope peaks for each m/z value. Due to the mass effect, the exchange of one atom by an isotope gives different m/z values for the iso­topologues, depending on the element. Therefore, depending on the resolution, the structure of the cor­responding isotope peaks can be complicated by the isotopic contri­butions of oxygen and hydro­gen


[7].

Table S 3: Isotopic abundancies of carbon, oxygen and carbon

| Element | Isotope (natural abundance / %) | | |
| --- | --- | --- | --- |
|  | Carbon C | Hydrogen H | Oxygen O |
| Main isotope | ^12^C (98.9355) | ^1^H (99.9885) | ^16^O (99.7576) |
| Isotope 1 (+1 neutron) | ^13^C (1.0645) | ^2^H (0.0115) | ^17^O (0.0383) |
| Isotope 2 (+2 neutrons) | -* | -* | ^18^O (0.2041) |
| *: not existing or negligible | | | |

## Resolution of isotopic peak patterns

Due to the oxygen and hydrogen atoms, even in case of simple compounds like e. g. gallic acid and ellagic acid, and only considering the first isotope peak, theore­ti­cal­ly three different ion masses can appear, having either one ^13^C (m/z 171.0249) or one ^17^O (m/z 171.0257) or one ^2^H atom (m/z 171.0278). Thus, theoretically, in case of a high intensity of the base peak, a more complex peak could be visible. However, the in­ten­sities of the two peaks which are due to ^2^H and ^17^O are considerably smaller than that of the first isotope peak, which is due to the higher abundancy of the ^13^C atom. Furthermore, the peaks are merged and the mea­sured isotope peak re­pre­sents the sum of these three isotopic peaks, since the separation of all isotope peaks would need a resolution R > 350,000. The lowest resolution required to separate isotopic peaks of ^13^C and ^2^H amounts to R > 57800 which is already significantly higher than the available resolution of 22,000. For the second and the third isotope peak the peaks be­come even more complex due to the increased number of possible isotopic combi­na­tions and the hi­gher content of ^18^O oxygen (0.204 %).

Since the isotopic peaks could not be resolved, for the calcu­la­ti­on of the inten­sities of the first and second isotope peak, all isotope combi­na­tions were con­sidered, those with one or more ^13^C atoms, which are contributing the most (1.08 %) to the total intensity, as well those as with ^2^H (0.016%), ^17^O (0.038%) and ^18^O (0.2%)


[7]. All cal­culations of ion masses and intensities were performed using the isotopic element masses and the electron mass given by Gross


[7]. For the cal­cula­tion of the theo­re­tical ratios of the isotopic peaks relative to the monoisotopistic peak, the formula given by Beynon


[3] was used and ex­tended to the third isotopic peak. For the isotopic peaks of GA and EA, the values provided in Table S 4 have been calculated.

Table S 4: Intensity ratios for the isotope peaks of GA and EA.

|  | First Isotope peak/ % | Second isotope peak/ % | third isotope peak/ % |
| --- | --- | --- | --- |
| GA | 7.81 % | 1.289 | 0.085 |
| EA | 15.48 % | 2.757 | 0.304 |

The calculation of the ion masses of all species and their fragments, as well as the m/z values and intensity ratios of the isotope signals, were performed based on their chemical formulae using Microsoft Excel 2019®.

## Range of intensities used for the determination of the ion ratios

The intensity traces (in main article: Figure 3, 5, 8, 10, 12 and 15) given by MassLynx for a given m/z value comprise ions in the range m/z - 0.025 to m/z + 0.025. The following pictures show the peaks in this range for the signals studied in this work. Figure S 2 shows the peaks around m/z 169 in the range m/z-0.5 to m/z+0.5.

Figure S 2: Peaks in the region 168.5 to 169.5. In the range 168.985 (169.01 - 0.025) to 169.035 (169.01 + 0.025), there are no other peaks.

## Identification of compounds by the tool “Elemental composition”

In some spectra a maximum fit confidence (%fit) of only 1% was obtained by MassLynx for a species with the formula C_14_H_5_O_8_ (EA-), whereas %fit of the composition C₁₇H₁O₆ was above 99%. According to the calculated value of DBI = 18, the neutral compound could theore­tically exist. However, this is not likely due to the very high number of carbon atoms relative to the number of hydrogen atoms which represents an ab­normally low hydrogen content.

For m/z 302 a species with the composition C_10_H_7_O_11_ was suggested by MassLynx with a fit con­fi­dence above 90%. However, although a value of DBE =8 does not exclude the existence of such a compound, it would have an unu­sually high oxy­gen content and a too low thermal stability. For the same reason, the species with compositions C_7_H_11_O_13_ (DBE =3), C_7_H_13_O_14_ (DBE =2) and C_7_H_9_O_13_ (DBE =4), C_13_H_10_O_5_ (DBE =10) suggested by MassLynx, were also considered as implausible.

None of the mentioned species was not found to be part of the background and none of the corresponding neutral compounds could be found in the literature.

It was concluded that the tool “Elemental composition” could not correctly determine the true composition because the minor isotope peak pattern of the present species was altered by signals of other species. Since the formulae C_10_H_7_O_11_, and C_17_H_1_O_6_ were typical sug­gesti­ons in many evaluations in this work, the reason for such suggestions is briefly explained in the following. The suggestion of a higher number of carbon atoms in the molecule by Mass­Lynx arises from the enhanced signal intensity of the first minor isotope peak which is due to overlap with another species at m/z 302, which leads to the pro­posed composition C_17_H_1_O_6_. Conversely, the suggestion of a high oxygen content, such as C_10_H_7_O_11_, can be explained by the interference at the second minor isotope peak of the species at m/z 301 with another species at m/z + 2. On the one hand, for a small molecule (below C90), substantially higher signals at m/z+1 and m/z+2 due to ^13^C atoms


[7] can be ruled out. On the other hand, in the absence of such inter­fe­rence, a high signal at m/z+2 can only be interpreted as resulting from a higher ^18^O content, since the minor isotope signal at m/z 303 is mainly increased by the number of ^18^O atoms of a molecule (due to the content of ^18^O of 0.2%). Because such for­mu­lae are inconsistent with known organic struc­tures, they most likely result from a mis­assign­ment, caused by isotope interference as explained, and peak overlap due to insuf­ficient resolution which prevents the isotope peaks from being differentiated from the monoisotopic or minor isotope peaks of other species at m/z 302 and m/z 303. This has already been shown for GA in this work and is fur­ther demon­strated by ASAP measure­ments with ellagic acid (main document section 3.6), which revealed the same issue.

## Study of the isotopic ratios in the range m/z 301 to 306

The signals indicated that two species were present with ions at m/z 303 and 304. Since the minor isotope peak of the small signal at m/z 301 has a negligible signal intensity at m/z 303, the signal intensity of the latter must be entirely assigned to the normal anion of MP. The signal at m/z 304 is approximately as high as that at m/z 303. As expected for the signal of a radical anion at m/z 304, minor isotope peaks were situated at m/z 305 and 306. Thus, assuming that no other species than m/z 303 and 304 was present, the intensities of the signals at m/z 303 and 304 and their minor isotope peaks at m/z 304 to 306 should be as calculated in Table S 5.

Table S 5: Signal intensities of the species at 303 to 305 with their corresponding minor isotope peaks (i.p.) in the ASAP measurement of the sample TA ink 2.

|  | m/z 303 | m/z 304 | m/z 305 | m/z 306 |
| --- | --- | --- | --- | --- |
| m/z 303 | 8.4E8 | 1.31E8 (i.p.1: 15.5%) | 2.34E7 (i.p.2: 2.8%) | 2.56E5 (i.p. :3: 0.3%) |
| m/z 304 | - | 7.09E8 (difference) | 1.10E8 (i.p.1: 15.5%) | 2.00E7 (i.p. 2: 2.8%) |
| observed |  | 8.4E8 | 2.40E8 | 4.0E7 |
| difference | 8.4E8 | - | ~1.1E8 | ~2.0E7 |
|  | | | | |

The calculated values are of the same order of magnitude as the measured intensities but the measured values are considerably higher for an unknown reason. In total, the observed inten­sities corresponded well to the statement that the anion and the radical anion of MP (molar mass: 304 g/mol) were present.

# **ESI measurements of tannin**

In contrast to insoluble inks, TA can be measured in solution and analyzed as a whole molecule by ESI to determine EA and the general compo­sition of the molecule. Except for Fu et al.


[6], who suggested TA as a calibration substance and pub­lished a nano-ESI measurement with potas­sium salt in the positive mode, no ESI measurement of TA could be found. They obtained peaks in the range of m/z 371.0368 to 1739.1169, which were separated by a mass difference of 152.0. No significant peaks were found in the region m/z 300 to 305. Fu et al.


[6] interpreted the ion masses in the spectrum as fragments of tannin and observed a maximum molecule size of m/z 1699 g/mol. A question which was not addressed by Fu et al.


[6], who utilized a tannin product (as is) from Sigma Aldrich, was the composition of the product used for the experiment. Since TA is mostly a mixture of several com­ponents and no separation has been performed before the ESI measurement, the large distribution of ion masses could be due either to fragmentation of TA in the MS or due to the presence of a mixture of different substances (or both). This does not change its usability ­as a calibrant, as suggested by Fu et al.


[6], but has consequences for the interpretation of the spectrum. Since TA is pro­duced directly from natural resources such as gall nuts, it is clear that, without chromatographic sepa­ration^^[[1]](#footnote-1)^^, it cannot be a uniform product. Furthermore, according to these findings, pentagallo glucose given in the review


[9] as formula for tannin, is not the compound with the highest number of galloyl groups of this complex mixture. Although a chromatogram of the utilized commercial TA product was not available, the HPLC chromatogram^^[[2]](#footnote-2)^^ of a similar TA product from Sigma-Aldrich might give an impression of the complexity of the com­position of commercial tannin products. The most expen­sive - and thus best purified – pentagallo tannin product, “tannin” from Sigma Aldrich, gave the following HPLC-chroma­togram^2, 3^ (Figure S 3) which proves that it should have contained more than 20 compounds at consi­derable percentages despite the purification process for its production.


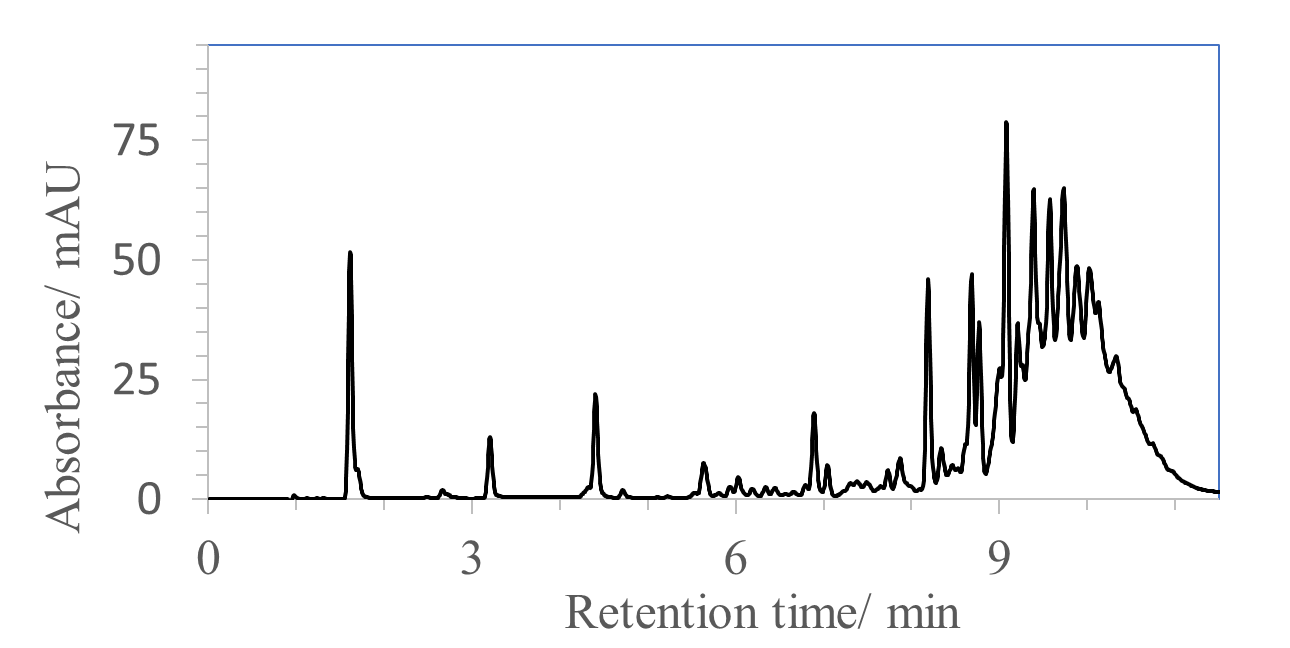


Figure S 3: HPLC chromatogram of a TA sample^^[[3]](#footnote-3)^^


[11, 12]

with absorbance in milli absorbance units (mAU).

Other, more affordable products are likely to be equally complex if not more than this product. In this study, TA was analyzed by ESI in the negative ionization mode without the addition of adduct forming agents. To confirm the absence of EA and related compounds in the TA, used for the production of the inks, or estimate their content, the mass spectrum was evaluated. Apart from the signal of the GA anion at m/z 169 and a small EA signal at m/z 301, in principle two signal groups could be distin­guished which were both characterized by a constant mass difference of Δ(m/z) 152, as also found by Fu et al.


[6]. An ion mass of m/z 1699.192, corresponding to that observed by Fu et al.


[6] and representing a deprotonated TA molecule with ten galloyl groups (main article, Figure 1), was also found in this work as part of the principal signal group with the most prominent signals (Figure S4).

Figure S4: Mass spectrum obtained from an ESI-measurement of TA solution (2.3E-5 mmol) in the negative ionization mode Table7, Exp. 9), with magnification of the highest ion masses, showing the typical isotopic pattern of the signals.

Beginning for clarity with the highest masses of the principal group, the latter were ranging from small signals at m/z 1851.192 and 1699.168 to m/z 332.26 (with their corresponding isotope peaks). Such ion masses cor­respond to galloyl glucose molecules with a varying number of galloyl groups from 11 (at m/z 1851.192) to 1 (at m/z 331.0648). Thus, the tannin contained probably mostly deca­gallo glu­cose and, at least in part, undeca­gallo glucose. In agreement with the results of Fu et al.


[6], no evidence for the presence of ellagic acid units within the TA molecule was observed. Owing to their low inten­sity relative to the background signals, signals with m/z values below m/z 635 could only be assigned by calculating their ion masses in relation to the more intensive peaks.

Most signals of the first group with high signal intensities were accompanied on the right by a second group of smaller signals, ranging from m/z 657.071 to 1721.1544. The con­stant difference of Δ(m/z) ~22 observed in these signals relative to the first group (mass M - 1) can be attributed to the loss of an additional hydrogen from the anions and the subsequent attachment of a sodium ion (Na^+^, M = 23), li­kely originating from the calibration with sodium formate performed shortly before, Accordingly, the signal at m/z 1721 can be assigned to a single charged anion,­ formed from a doubly deprotonated TA molecule and an added sodium ion ((M - 1) -1 +23), the positive charge of the sodium ion compen­sa­ting one negative charge. An evaluation of the relative intensities of the signals at m/z 301 (0.16 % of total intensity), cor­res­ponding to the content of EA, and those corresponding to TA (6.2 % of total in­ten­sity) of the ESI measurement (Figure S4) showed that the content of EA was too small to cause the important signals obtained with TA and TA inks in ASAP measurements.

# **Break down products of gallic acid**

Using higher collision energies of 30 eV, apart from the given species, further smaller break-down products (< m/z 169) could be found with ASAP measurements in the MSMS mode (main article, Table7, Exp. 6). Apart from the pyrogallol ion at m/z 125, there were signals at m/z 107, 79, 81 (Figure S5) as well as m/z 97 and 69 (Figure S6). The interpretation is given in the main article (Table 3).

.

Figure S5: Mass spectrum obtained at 5.294 min from the chromatogram of the ASAP measurement of GA in the negative mode (in main article Table7, method 6).

Figure S6: Mass spectrum obtained at 5.920 min from the chromatogram of the ASAP measurement (MSMS mode) of GA in the negative ionisation mode (in main article Table7, method 6).

1. **Charge envelope of ions**

In the mass spectra of the ASAP measurement with a collision energy of zero (main article, Table 7, Exp. 8) for the signals of the ions at m/z 169, 321 and 339, in addition to the isotope peaks at m/z + 1, m/z + 2 etc., signals were obtained at non-in­teger numbers. Whereas For highly charged pro­tein mole­cu­les


[8], it is known that the ions have m/z values with similar decimal places. The latter are the conse­quen­ce of the division of the ion mass of the molecule by the charge number of the anion, the general formula being given by eq. 2.

| $\frac{m}{z}=\left[ \frac{M-z*m\left( H \right)}{z} \right]$  M: molar weight: z: charge number | eq. 2 |
| --- | --- |

Using eq. 2 and two adjacent m/z values, the charge number z was calculated and its rounded value used with the m/z value to obtain M. The m/z values of multiply charged ions are typically separated by several m/z units while isotopologues exhibit nearly identical nominal m/z values with minor deci­mal differences. Isotope peaks of the multicharged ions appeared at the m/z value of the monoisotopic singly charged ion with approximately fixed increments (m/z + 0.25, m/z + 0.5 etc.) as well as at the corresponding isotope peaks with similar decimal places. In the following the additional signals around the base peak and the isotope peaks for the considered ions are discussed.

- 1. **Ion at m/z 321**

Except for the peak at m/z 321.0229 (M = 322 g/ mol) and its isotope peaks at m/z 322.0283, 323.0287, additional peaks were present, e.g., at m/z 321.2542, 321.4839 and 321.75 and in part also at the m/z values of the isotope peaks (322.25; 322.50, etc.) (Figure S7).

Figure S7: Traces of the ion signals at m/z 301 to 305 obtained from the chromatogram of an ASAP measurement of GA powder in the negative ionization mode (main article Table 6, Exp. 7) .

Using the formula eq. 2, a charge number of z = 4.25 was calculated. Thus, the m/z values would corres­pond to that obtained with a fourfold charged tetra­mer of the ion at m/z 321 and its isotope signals. Since the m/z value of the monoisotopic species of the tetramer matches with that of the single charged anion, only the isotope peaks of it are detected at m/z +0.25, m/z + 0.5 and m/z + 0.75. All m/z values of the isotope peaks of the single charged ion and the m/z values of the tetramer between m/z 321 and 322 are given in Table S 6.

Table S 6: Ions of the charge envelope at 321.023

| Molecule | m/z calculated | Measured m/z  (error in mDa) | m/z calculated theoretically | From measurement  (error in mDa) |
| --- | --- | --- | --- | --- |
|  | Monomer | | Tetramer | |
| Neutral theoret. | 322.0325 | 322.303 (2.2) | 1288.1211 | 1288.0430 (87) |
| Anion | A^-^ | | A^4-^ | |
| Monoisotopic | 321.0252 | 321.0230 (2.2) | 321.0252 | 321.0229 (1.6) |
| 1st sat. | 322.0286 | 322.0280 (0.6) | 321.2760 | 321.2542 (21.7) |
| 2nd sat. | 323.0319 | 323.0300 (1.9) | 321.5269 | 321.4839 (43.1) |
| 3rd sat. | 324.0353 | 324.0315 (3.8) | 321.7777 | 321.7450 (63.2) |
|  | | | | |

There were further, smaller peaks, e.g. at 321.166, 321.40 and 321.90, presumably corresponding to ions with higher charges. However, it was out of the scope of this article to analyze such peaks in detail.

- 1. **Charge envelope of m/z 339**

The composition C_14_H_11_O_10_ yields an m/z value of 339.034 with isotope signals at m/z 340.038, 341.041 and 342.043. Additional signals were at m/z 339.28, 329.54 and 339.79 next to the single charged anion and at the m/z values of its corresponding isotope peaks at 340 and 341. Formula eq. 2 was used to calculate the molar weight of the neutral species (Table S 7).

Table S 7: Ions of the charge envelope at 339.034

| Molecule | m/z calculated | Measured  (error in mDa) | m/z calculated theoretically | From measurement  (error in mDa) |
| --- | --- | --- | --- | --- |
|  | Monomer | | Neutral tetramer | |
| Neutral molecule | 340.0416 | - | 1360.166 | 1360.113 (53) |
| Anion | A- | | A4- | |
| Monoisotopic | 339.0343 | 321.0340 (0.3) | 339.0343 | 321.0340 (0.3) |
| 1st sat. | 340.0377 | 340.0380 (0.3) | 339.2851 | 339.2718 (13.3) |
| 2nd sat. | 341.0410 | 341.0406 (0.4) | 339.5360 | 339.5076 (28.4) |
| 3rd sat. | 342.0444 | 342.0430 (1.4) | 339.7868 | 339.7454 (41.8) |
|  | | | | |

- 1. **Discussion of charge envelopes for the given species**

On one hand, the measured m/z values give patterns which are typical for multiply charged ions and the formation of the latter is plausible. Due to the collision energy of zero in these measurements, molecular associates are more stable than under the usually utilized conditions. Furthermore, a tetra­mer with a higher charge is possible because charges of the ions are relatively separated by the benzene rings. On the other hand, the measured values deviated from those calculated considerably more than the value m/z of the monocharged anion. However, it is known that the measurement error for multiply charged species generally is higher than for monocharged anions. The deviations are likely to be due to a non-adequate cali­bra­tion, detector linearity issues etc. Furthermore, the measured m/z values could not be attri­buted unambiguously to the compositions cor­responding to the hypothe­sized spe­cies. The latter issue might be related to the considerable difference of the calculated m/z to the measured m/z which leads also to misplaced positions of the isotope peaks and a misassignment by MassLynx.

# **Traces of ions observed with EA**

Figure S8 shows the signal courses at m/z 301, 302 and 303 during the ASAP measurement of EA. The intensities of the isotope peaks corresponded approximately to those calculated theoretically (15.5% and 2.8 %) for the first and the second isotope peak, respectively, at the beginning of the EA peak. At lower and higher retention times the ratio deviated significantly from the theoretical values.


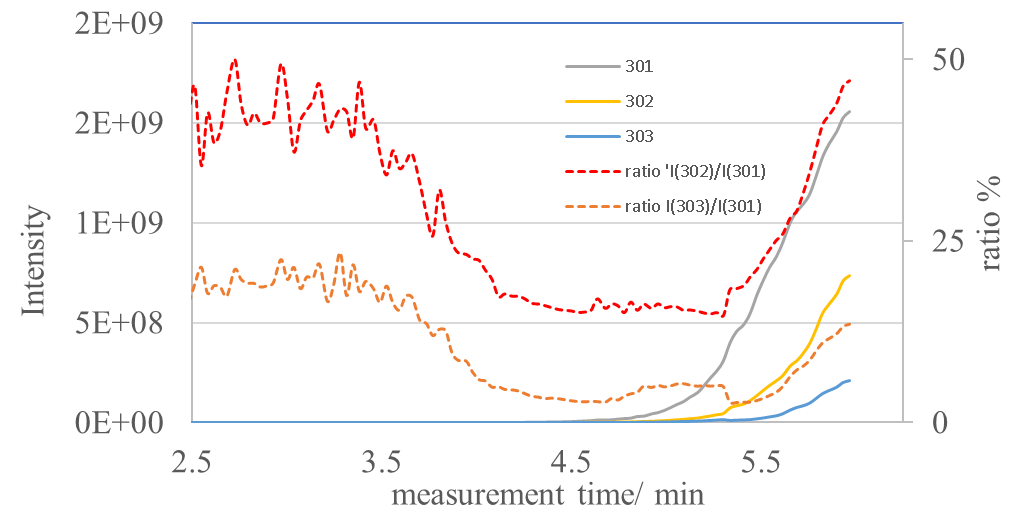


Figure S8: Intensity courses of the ratios of the intensities of the signals at m/z 302 and 303 to that of 301 obtained from an ASAP measurement of EA in the negative ionisation mode (Table 7, method 9).

# **Synthesis of ellagic acid**

The formation of EA and possible precursors from two GA molecules by condensation of the carboxyl group with a hydroxyl group in meta position is similar to the synthesis of EA by dimerization of GA after oxidative coupling and lactone ring formation


[1]. It has been synthesized, e.g., from gallate and by catalytic coupling of aryl compounds


[1, 2, 5, 10], but also easily in one step from ethyl gallate


[2, 4, 10]. Literature indicates that the synthesis of EA is quite challenging due to the necessity to couple two rings in the presence of easily oxidizable phenolic groups and due to solubility problems of EA.

# **References**

[1] Agrawal, O.D. and Kulkarni, Y.A. 2020. Mini-Review of Analytical Methods used in Quantification of Ellagic Acid. *Reviews in Analytical Chemistry*. 39, 1 (Jan. 2020), 31–44.

[2] Alam, A. and Tsuboi, S. 2007. Total synthesis of 3,3′,4-tri-O-methylellagic acid from gallic acid. *Tetrahedron*. 63, 42 (Oct. 2007), 10454–10465.

[3] Beynon, J.H. 1960. Mass spectrometry and its applications to organic chemistry. Elsevier Publishing Company. 294–302.

[4] Cajnko, M.M. et al. 2024. Hydroxide-Catalyzed Ellagic Acid Synthesis from Biobased Ethyl Gallate Using Deep Eutectic Solvents. *ACS Sustainable Chemistry &amp; Engineering*. 12, 10 (Feb. 2024), 4007–4016.

[5] Daley, S. and Downer-Riley, N. 2020. The biomimetic synthesis of balsaminone A and ellagic acid via oxidative dimerization. *Beilstein Journal of Organic Chemistry*. 16, (Aug. 2020), 2026–2031.

[6] Fu, L. et al. 2019. Tannic Acid: a Novel Calibrator for Facile and Accurate Mass Measurement of Electrospray Ionization Mass Spectrometry. *Journal of the American Society for Mass Spectrometry*. 30, 8 (Jun. 2019), 1545–1549.

[7] Gross, J.H. 2017. *Mass spectrometry*. Springer.

[8] Mann, M. et al. 2001. Analysis of Proteins and Proteomes by Mass Spectrometry. *Annual Review of Biochemistry*. 70, 1 (Jun. 2001), 437–473.

[9] Melo, M.J. et al. 2022. Iron-gall inks: a review of their degradation mechanisms and conservation treatments. *Heritage Science*. 10, 1 (Sep. 2022).

[10] Pujol, M.D. et al. 2022. Convenient Synthesis of Ellagic Acid from Methyl Gallate and SARS-CoV-2 3CLpro Antiviral Activity. *Synthesis*. 55, 04 (Sep. 2022), 657–662.

[11] SigmaAldrich 2023. Tanninsäure. Sigma Aldrich.

[12] Sigma-Aldrich 2025. Analysencertifikat. Merck CoKG.

1. Special products of digallo tannins with defined composition – such as Hamamelis tannin” are expensive due to the necessary chromato­graphic separation at preparative scale (5 mg >100 Euro). [↑](#footnote-ref-1)
2. HPLC chromatogram of a decagallo tannin (Pharmacopeia (USP) Reference Standard): Conditions: Column Ascentis 90A C18: Length 100 diameter:4.6 mm, 2.7μm particle diameter, gradient with phase A: water (0.1% TFA)

   B: methanol (t=0: 90 % A to t = 12 min 45% A. Flow rate: 1 ml/ min. injection volume 1.0 μl, DAD detector (270 nm). [↑](#footnote-ref-2)
3. Reproduction with the permission from Sigma Aldrich. [↑](#footnote-ref-3)
